# Supplementary material for: Lnc-TALC promotes O6-methylguanine-DNA methyltransferase expression via regulating the c-Met pathway by competitively binding with miR-20b-3p
Source: Nat Commun. 2019 May 3;10:2045. doi: 10.1038/s41467-019-10025-2 (PMC6499807; doi:10.1038/s41467-019-10025-2)
Supplement: Supplementary file 1 — Supplementary Information [file 41467_2019_10025_MOESM1_ESM.pdf]

SUPPLEMENTARY INFORMATION

Lnc-TALC promotes O^6-methylguanine-DNA methyltransferase expression via regulating the c-Met pathway by competitively binding with miR-20b-3p

Wu et al.

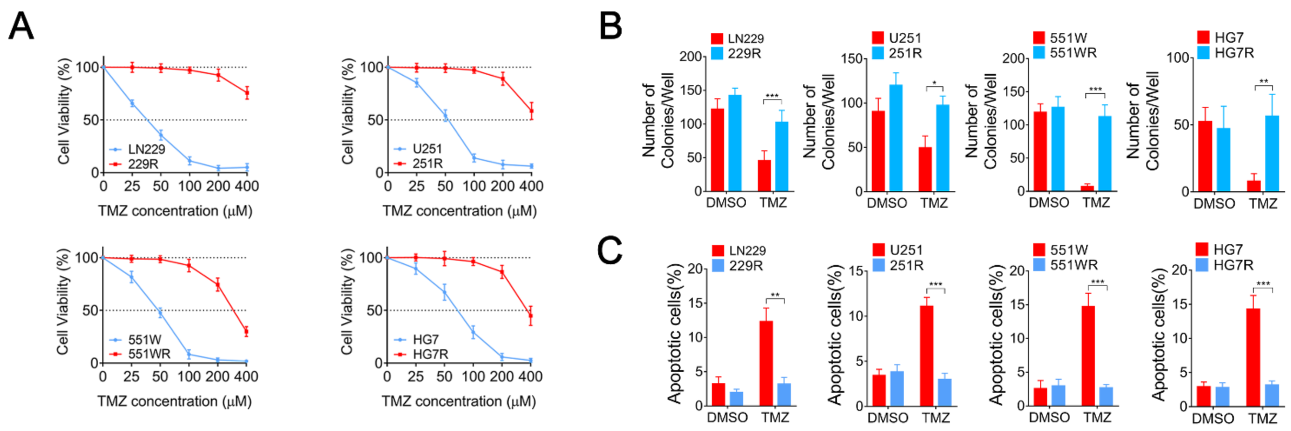

Supplementary Figure 1. The establishment of TMZ-resistant GBM cells.

A. CCK-8 assay of TMZ-resistant and parental GBM cells upon TMZ treatment at the indicated concentrations for 72 h (n=3).

B. Colony formation assay of TMZ-resistant and parental GBM cells upon TMZ treatment (100 μM) in a 6-well dish (300 cells per well) for 11 days (n=3). Representative images and the average number of colonies are shown.

C. Flow cytometric analysis of apoptosis in TMZ-resistant and parental GBM cells upon TMZ treatment (100 μM) for 72 h (n=3). Representative images and the average number of apoptotic cells are shown. Data are presented as the mean ± S.D. P value was determined by Student's t test. Significant results were presented as NS (non-significant), *$P < 0.05$, **$P < 0.01$ or ***$P < 0.001$.

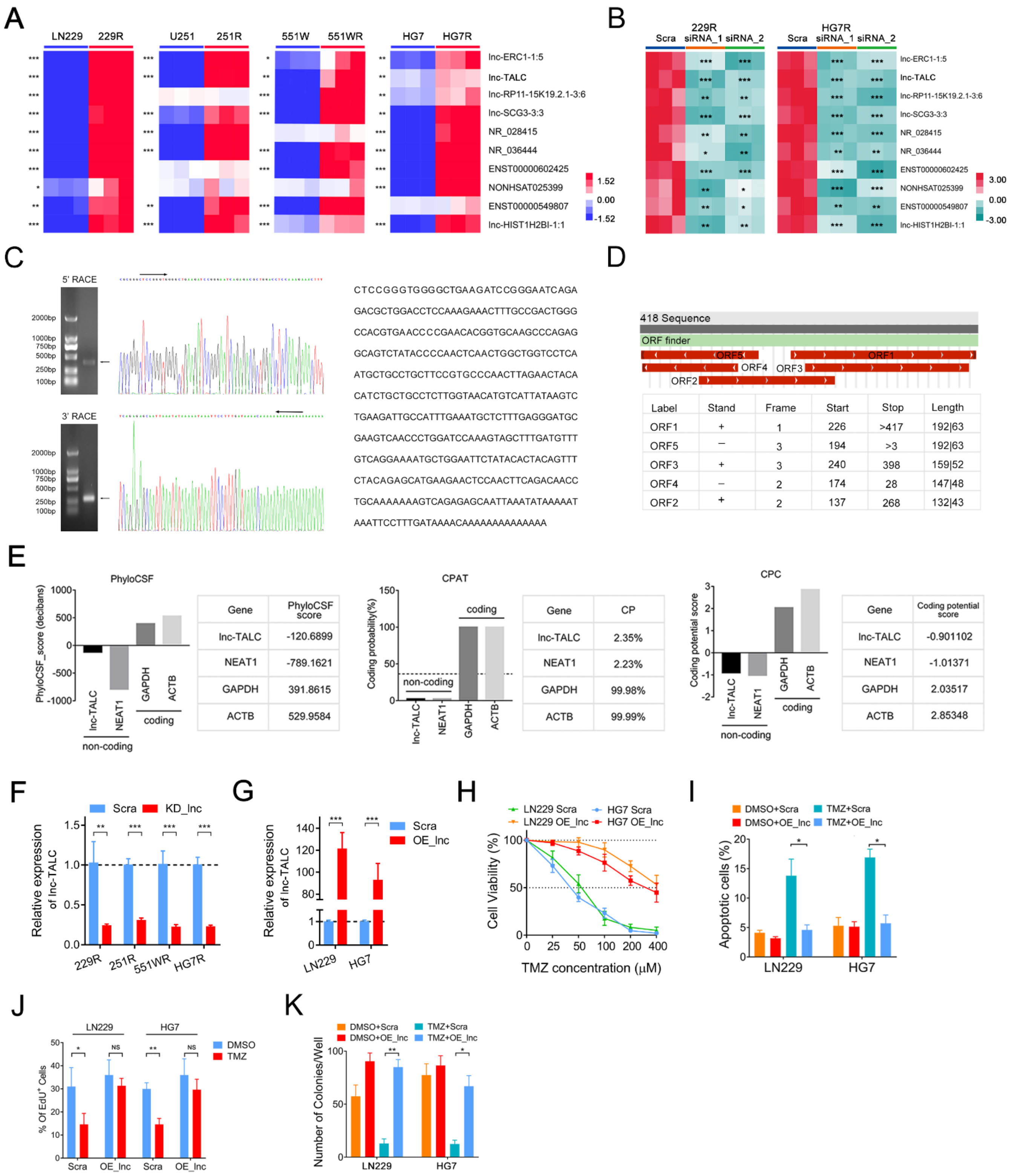

Supplementary Figure 2. The identification and association with TMZ resistance of lnc-TALC in GBM cells.

A. qRT-PCR analysis of the top 10 upregulated lncRNAs in TMZ-resistant and parental GBM cells.

B. qRT-PCR analysis validated the knockdown efficiency of the top 10 upregulated lncRNAs in TMZ-resistant GBM cells (n=3).

C. Left: Representative images of PCR products from the 5'RACE or 3'RACE procedure. The major PCR product is marked by an arrow. Middle: Sequencing of PCR products revealed the boundary of lnc-TALC

sequences. Right: The nucleotide sequence of full-length lnc-TALC.

D. Prediction of putative proteins encoded by lnc-TALC using ORF Finder.

E. Left: Coding potency of lnc-TALC sequence was analyzed using PhyloCSF. Middle: Coding potency of lnc-TALC sequence was analyzed using CPAT. Right: Coding potency of lnc-TALC sequence was analyzed using CPC. NEAT1 served as a control non-coding gene. GAPDH and β-actin served as control coding genes.

F. qRT-PCR analysis validated the knockdown of lnc-TALC in TMZ-resistant GBM cells.

G. qRT-PCR analysis validated the overexpression of lnc-TALC in parental GBM cells.

H. CCK-8 assay analysis revealed the effect of the lnc-TALC overexpression on parental GBM cells with TMZ treatment at the indicated concentrations for 72 h (n=3).

I. Cell apoptosis analysis revealed the effect of the lnc-TALC overexpression on the apoptosis of parental GBM cells with TMZ treatment (100 μM) for 72 h (n=3). Representative images and the average number of apoptotic cells are shown.

J. The EdU assay showed the effect of lnc-TALC overexpression on the DNA replication activity of parental GBM cells with TMZ treatment (100 μM) for 72 h (n=3). Representative images and the average number of EdU^+ cells are shown.

K. The colony formation assay showed the effect of the lnc-TALC overexpression on the growth of parental GBM cells with TMZ treatment (100 μM) in a 6-well dish (300 cells per well) for 11 days (n=3). Representative images and the average number of colonies are shown. Data are presented as the mean ± S.D. P value was determined by Student’s t test. Significant results were presented as NS (non-significant), $*P < 0.05$, $**P < 0.01$ or $***P < 0.001$.

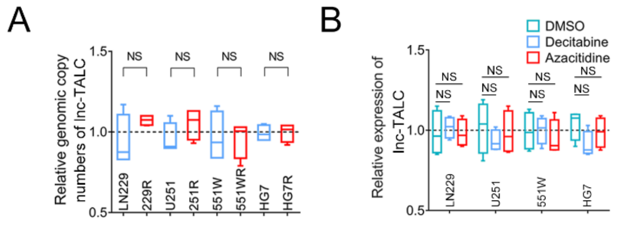

Supplementary Figure 3. Copy number and DNA methylation alterations of lnc-TALC.

A. qRT-PCR analysis of genomic copy numbers of lnc-TALC in TMZ-resistant and parental GBM cells.

B. qRT-PCR analysis of lnc-TALC in parental GBM cells treated with decitabine (0.5 μM), azacitidine (10 μM) or DMSO (0.3%). Data are presented as the mean ± S.D. P value was determined by Student's t test. Significant results were presented as NS (non-significant), *P < 0.05, **P < 0.01 or ***P < 0.001.

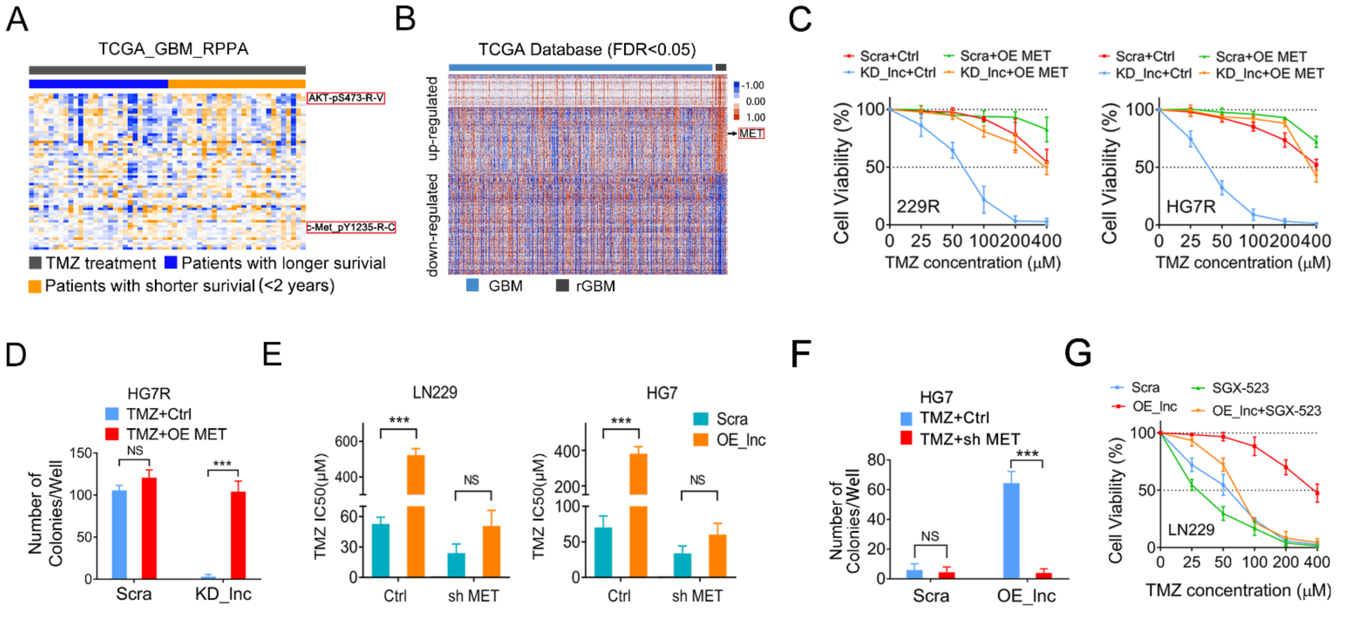

Supplementary Figure 4. C-Met affects lnc-TALC- induced TMZ resistance in GBM.

A. Differentially expressed proteins were selected according to the overall survival time in the TCGA database.

B. In the TCGA database, differentially expressed genes between primary GBMs and recurrent GBMs were selected.

C. CCK-8 assay analysis of the effect of MET overexpression on 229R and HG7R cells after knocking down lnc-TALC upon TMZ treatment at the indicated concentrations for 72 h (n=3).

D. Colony formation assay of the effect of MET overexpression on HG7R cells after knocking down lnc-TALC upon TMZ treatment (100 μM) in a 6-well dish (300 cells per well) for 11 days (n=3). Representative images and the average number of colonies are shown.

E. Determination of TMZ IC50 by CCK-8 assay analysis of the effect of sh-MET on LN229 and HG7 after overexpressing lnc-TALC (n=3).

F. Colony formation assay of the effect of sh-MET on HG7 cells after overexpressing lnc-TALC upon TMZ treatment (100 μM) in a 6-well dish (300 cells per well) for 11 days (n=3). Representative images and the average number of colonies are shown.

G. CCK-8 assay analysis of the effect of SGX-523 on LN229 cells after overexpressing lnc-TALC upon TMZ treatment at the indicated concentrations for 72 h (n=3). Data are presented as the mean ± S.D. P value was determined by Student's t test. Significant results were presented as NS (non-significant), *P < 0.05, **P < 0.01 or ***P < 0.001.

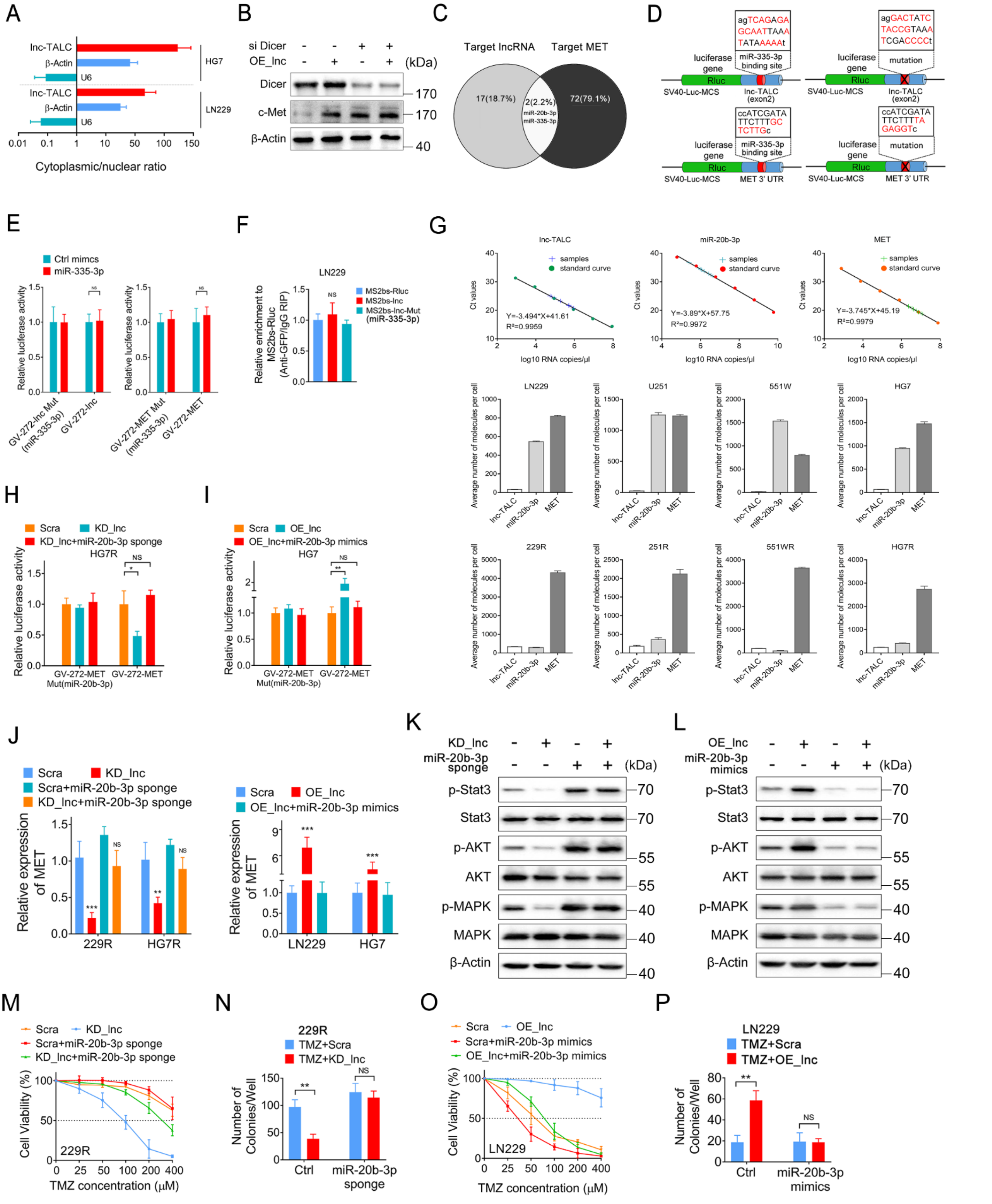

Supplementary Figure 5. The discovery and identification of miRNAs targeting lnc-TALC and MET.

A. qRT-PCR analysis of the cytoplasmic/nuclear ratio of lnc-TALC, β-Actin and U6 SnRNA.

B. Western blot analysis of Dicer and c-Met in LN229 cells transfected with LV-lnc-TALC, si Dicer or scramble.

C. Overlap of potential miRNAs targeting both lnc-TALC and MET 3’UTR regions.

D. The predicted miR-335-3p binding sites on lnc-TALC and the 3’UTR of MET.

E. Luciferase activity of GV-272-lnc-TALC or GV-272-MET upon transfection of control mimics and miR-335-3p in 229R (n=3).

F. MS2-based RIP assay with anti-GFP antibody in LN229 cells 72 h after transfection with MS2bs-Rluc, MS2bs-lnc-TALC or MS2bs-lnc-TALC-Mut (n=3).

G. Upper: The standard curves for molecular numbers of lnc-TALC, miR-20b-3p and MET based on plasmids (lnc-TALC or MET) or synthetic polynucleotide (miR-20b-3p) with known amounts. Middle: qRT-PCR analysis combined with standard curves revealed that the average molecular numbers per cell for lnc-TALC, miR-20b-3p and MET in parental GBM cells. Lower: The average molecular numbers per cell for lnc-TALC, miR-20b-3p and MET in TMZ-resistant GBM cells.

H. Luciferase activity of GV-272-MET in HG7R after knocking down lnc-TALC upon transfection of a miR-20b-3p sponge (n=3).

I. Luciferase activity of GV-272-MET in HG7 after overexpressing lnc-TALC upon transfection of miR-20b-3p mimics (n=3).

J. Left: qRT-PCR analysis of MET in TMZ-resistant GBM cells after knocking down lnc-TALC upon transfection of a miR-20b-3p sponge (n=3). Right: qRT-PCR analysis of MET in parental GBM cells after overexpressing lnc-TALC upon transfection of miR-20b-3p mimics (n=3).

K. Western blot analysis of the indicated proteins in 229R after knocking down lnc-TALC upon transfection with a miR-20b-3p sponge.

L. Western blot analysis of indicated proteins in LN229 after overexpressing lnc-TALC upon transfection with miR-20b-3p mimics.

M. CCK-8 assay analysis of the effect of miR-20b-3p sponge on 229R after knocking down lnc-TALC upon TMZ treatment at the indicated concentrations for 72 h (n=3).

N. Colony formation assays of the effect of a miR-20b-3p sponge on 229R after knocking down lnc-TALC upon TMZ treatment (100 μM) for 11 days (n=3). Representative images and the average number of colonies are shown.

O. CCK-8 assay analysis of the effect of miR-20b-3p mimics on LN229 after overexpressing lnc-TALC upon TMZ treatment at the indicated concentrations for 72 h (n=3).

P. Colony formation assays of the effect of miR-20b-3p mimics on LN229 after overexpressing lnc-TALC upon TMZ treatment (100 μM) for 11 days (n=3). Representative images and the average number of colonies are shown. Data are presented as the mean ± S.D. P value was determined by Student's t test or One-way ANOVA. Significant results were presented as NS (non-significant), *$P < 0.05$, **$P < 0.01$ or ***$P < 0.001$.

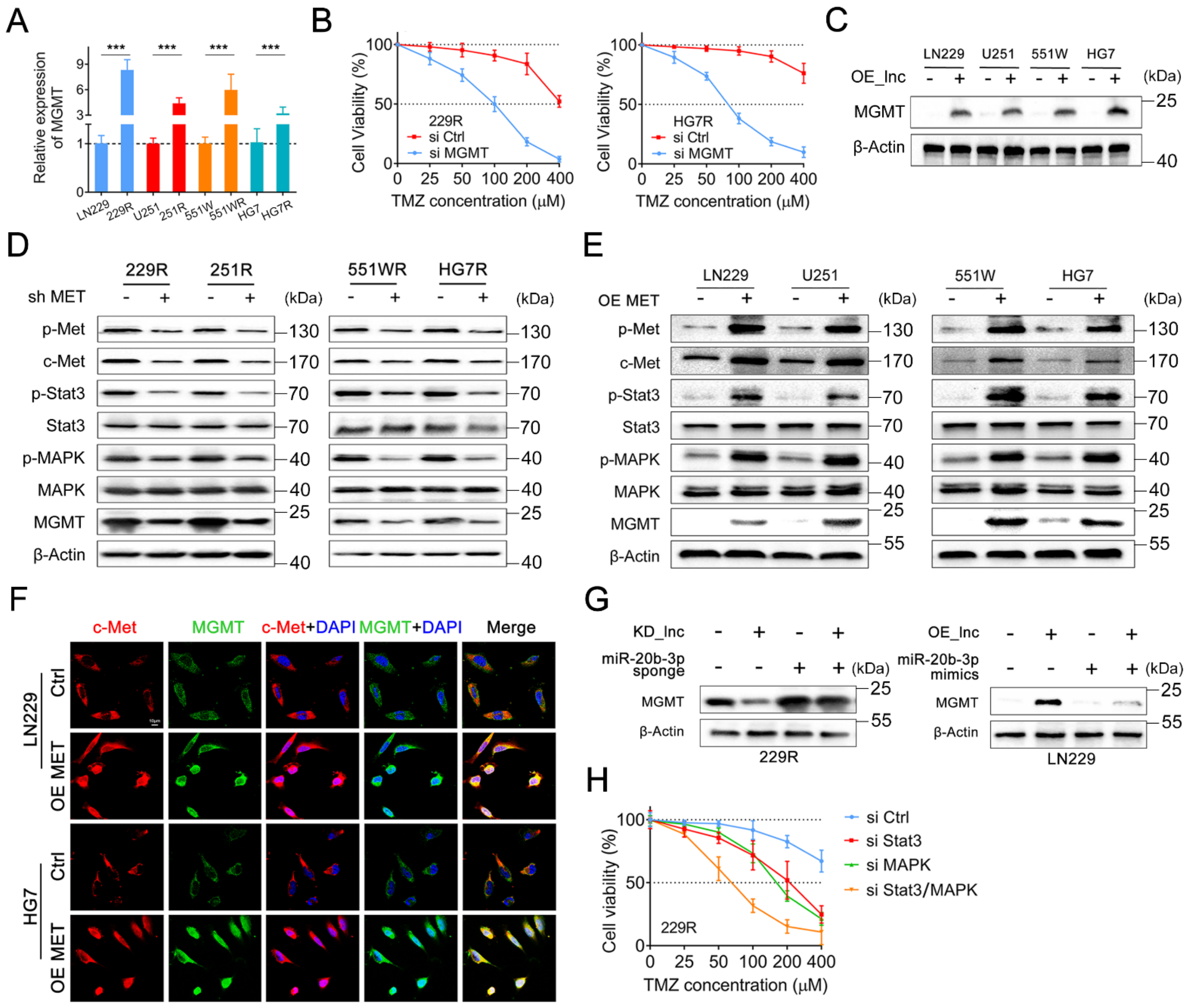

Supplementary Figure 6. C-Met is involved in MGMT-induced TMZ resistance of GBM.

A. qRT-PCR analysis of MGMT expression in TMZ-resistant and parental GBM cells.

B. CCK-8 assay analysis of TMZ-resistant GBM cells transfected with si-MGMT and si-control upon TMZ treatment at the indicated concentrations for 72 h (n=3).

C. Western blot analysis of MGMT expression in parental GBM cells transfected with LV-lnc-TALC.

D. Western blot analysis of the indicated protein in TMZ-resistant GBM cells transfected with sh-MET.

E. Western blot analysis of the indicated protein in parental GBM cells transfected with MET and scramble plasmid.

F. Immunofluorescence analysis of MGMT and c-Met in parental GBM cells transfected with MET and scramble plasmid. The nuclei were stained with DAPI. Scale bar=10 μm.

G. Western blot analysis of MGMT in 229R after knocking down lnc-TALC upon transfection with a miR-20b-3p sponge and in LN229 after overexpressing lnc-TALC upon transfection with miR-20b-3p mimics.

H. CCK-8 assay analysis of si-Stat3, si-MAPK, si-Stat3/MAPK and control in TMZ-resistant GBM cells upon TMZ treatment at the indicated concentrations for 72 h (n=3). Data are presented as the mean ± S.D. P value

was determined by Student's t test. Significant results were presented as NS (non-significant), $*P < 0.05$, $**P < 0.01$ or $***P < 0.001$.

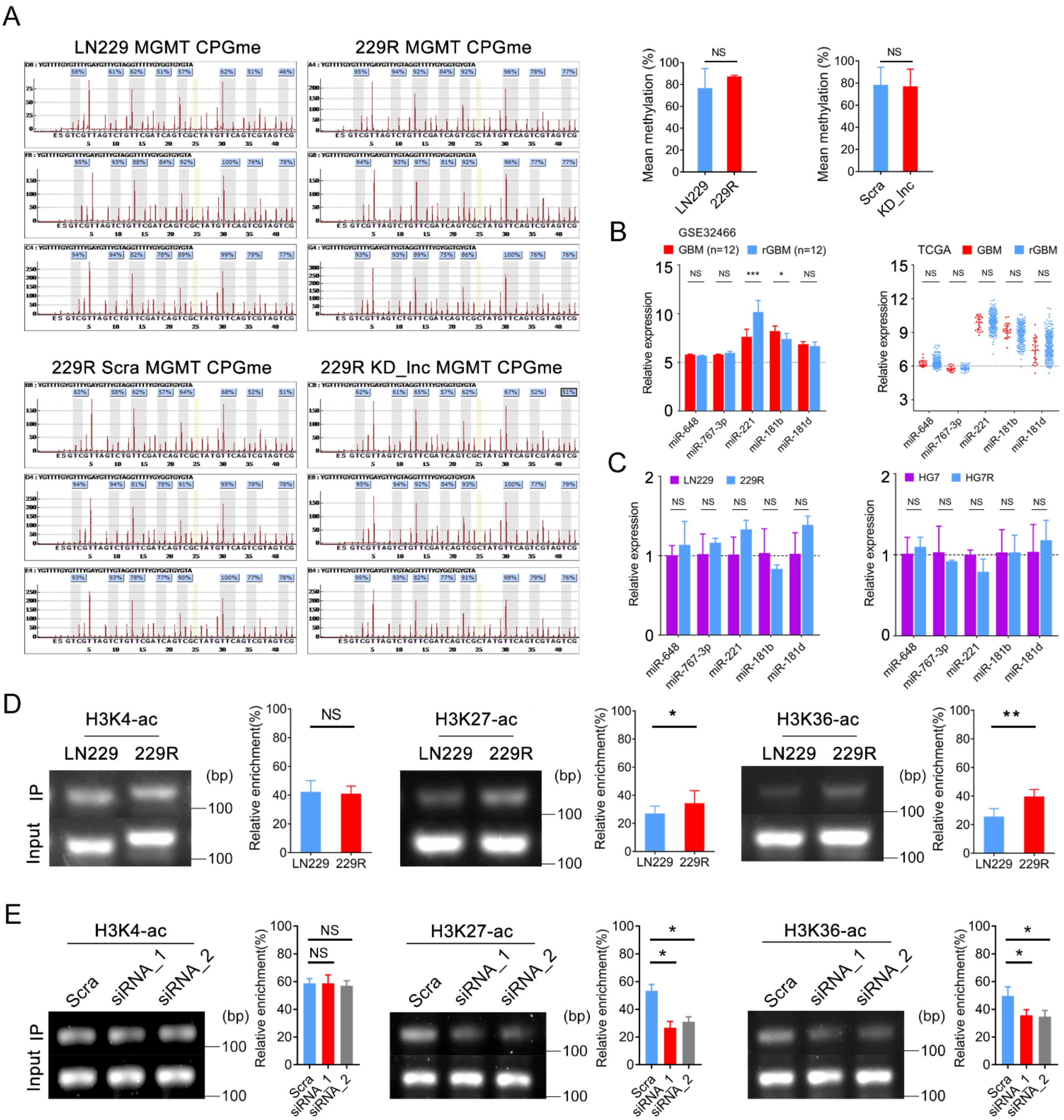

Supplementary Figure 7. The exploration of MGMT regulation in TMZ-resistant GBM cells.

A. Left: Validation of MGMT promoter methylation levels by pyrosequencing in LN229 and 229R cells (upper). Validation of MGMT promoter methylation levels by pyrosequencing in 229R after knocking down lnc-TALC (lower). Representative images and the average levels of MGMT promoter methylation (right) are shown.

B. The indicated relative miRNA expression levels were analyzed in the GSE32466 (left) and TCGA (right) databases.

C. qRT-PCR analysis of the indicated miRNA in parental and TMZ-resistant GBM cells.

D. ChIP assay of the enrichment of H3K4ac, H3K27ac and H3K36ac in the MGMT promoter region normalized to IgG in LN229 and 229R GBM cells (n=3).

E. ChIP assay of the enrichment of H3K4ac, H3K27ac and H3K36ac in the MGMT promoter region normalized to IgG in 229R GBM cells transfected with si-Stat3 or scramble. Data are presented as the mean ± S.D. P value was determined by Student's t test. Significant results were presented as NS (non-significant), $*P < 0.05$, $**P < 0.01$ or $***P < 0.001$.

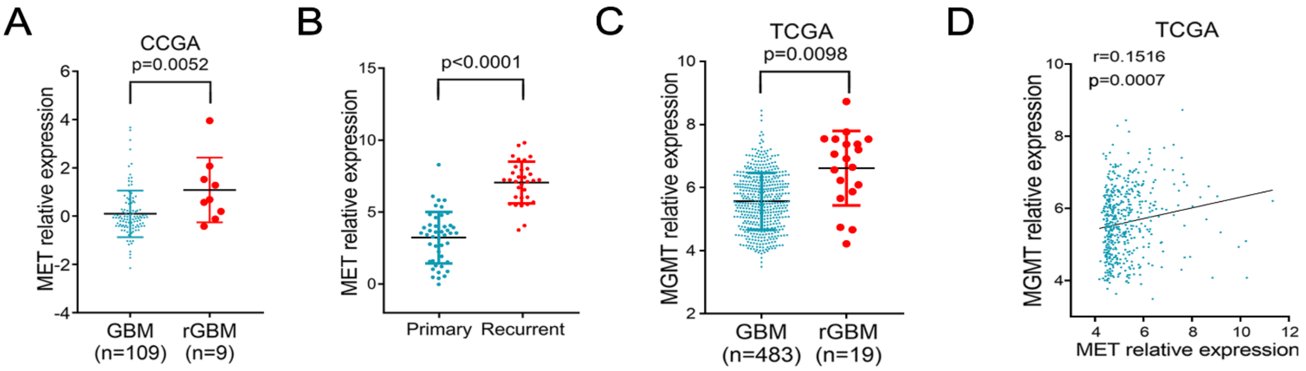

Supplementary Figure 8. MET and MGMT are responsible for the recurrence of GBMs patients.

A. MET relative expression was analyzed between primary and recurrent GBMs in the CGGA database.

B. qRT-PCR assay analysis of MET relative expression between primary and recurrent GBM samples.

C. MGMT relative expression was analyzed between primary and recurrent GBMs in the TCGA database.

D. Pearson correlation analysis between MGMT and MET expression levels in the TCGA database. Data are presented as the mean ± S.D. P value was determined by Student's t test or Pearson's correlation test. Significant results were presented as NS (non-significant), $^*P < 0.05$, $^{**}P < 0.01$ or $^{***}P < 0.001$.

Figure 2G

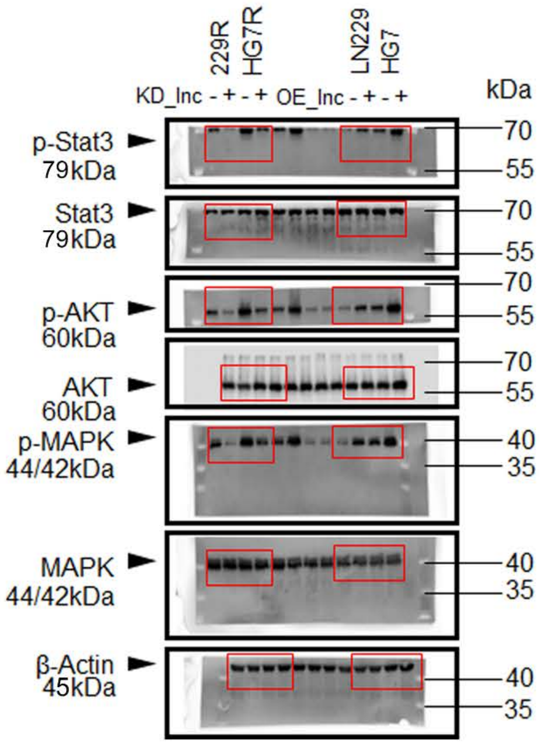

Figure 3D

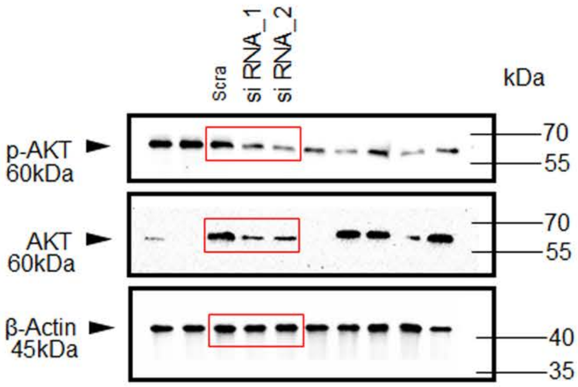

Figure 3E

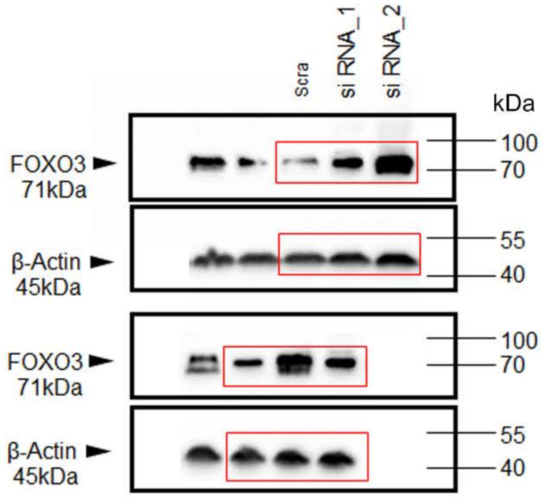

Figure 3F

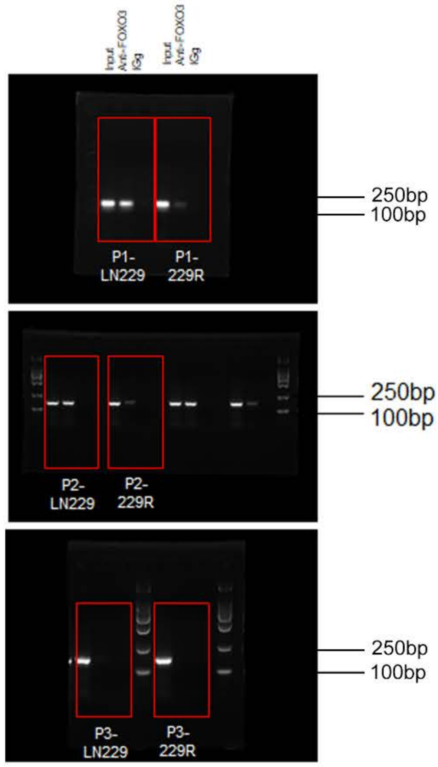

Figure 4B

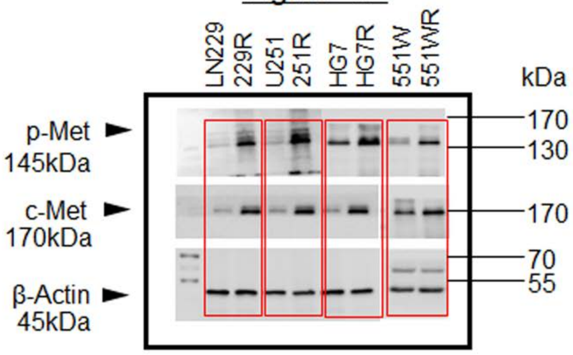

Figure 4D

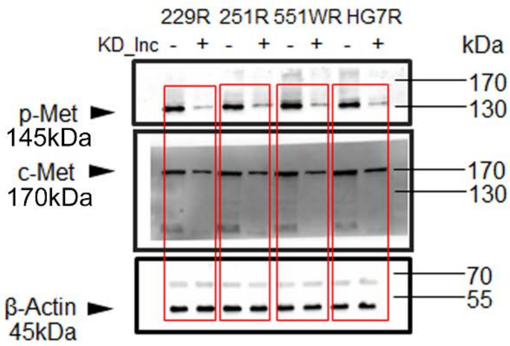

Figure 4F

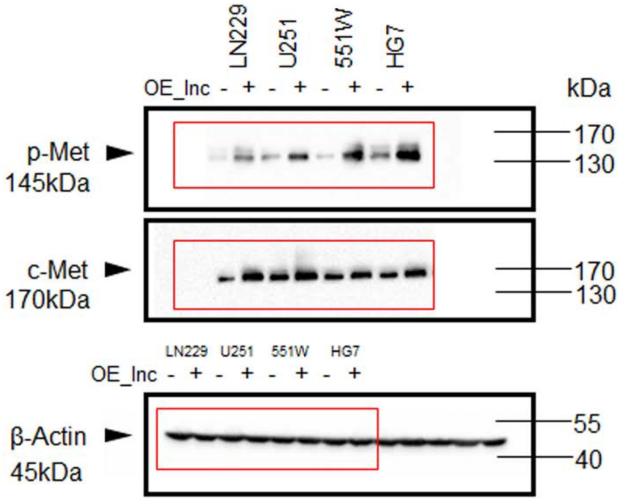

Figure 4H

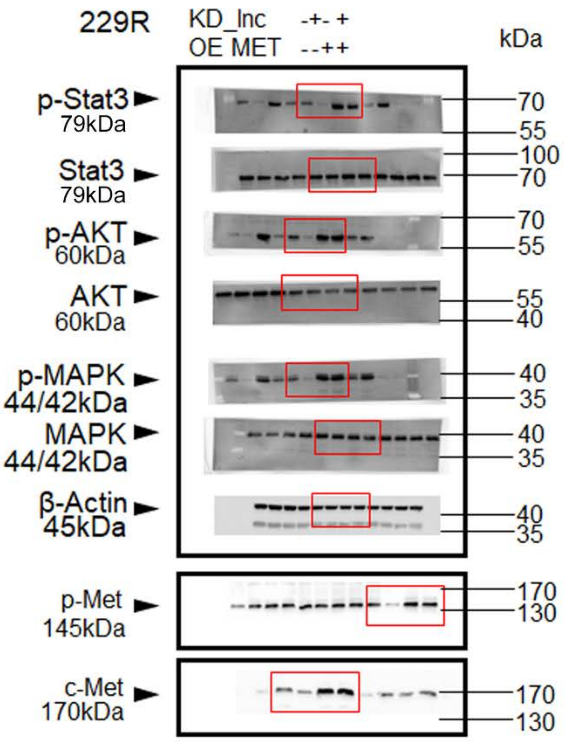

Figure 4J

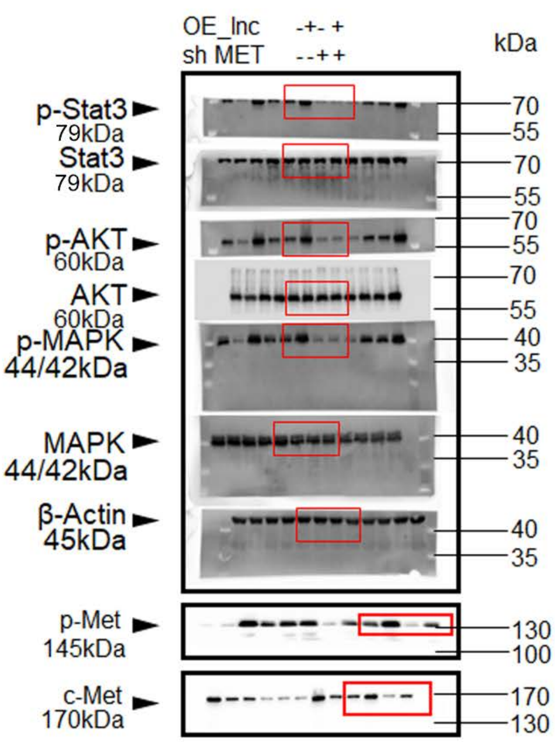

Figure 4L

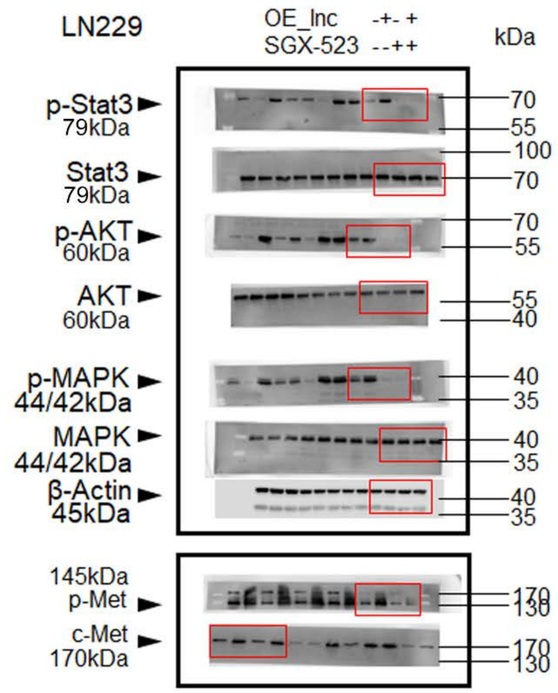

Figure 5G

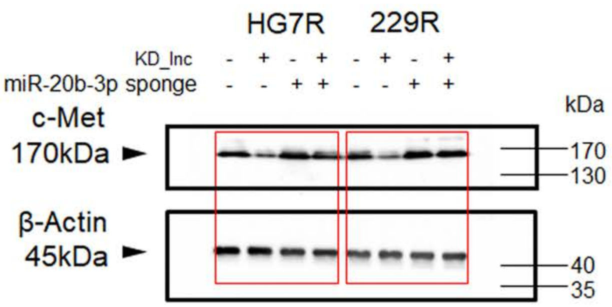

Figure 5H

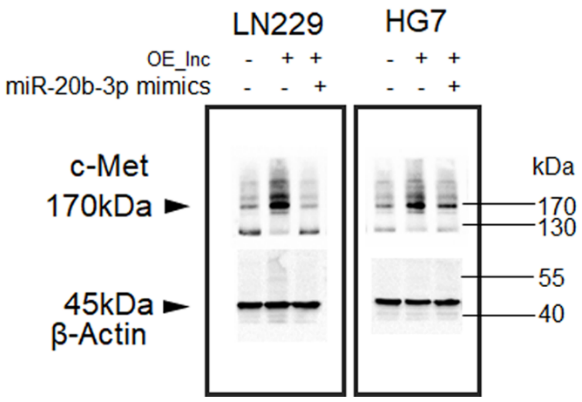

Figure 6A

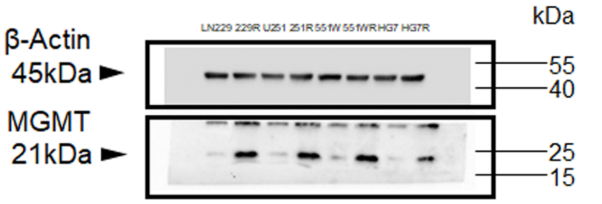

Figure 6D

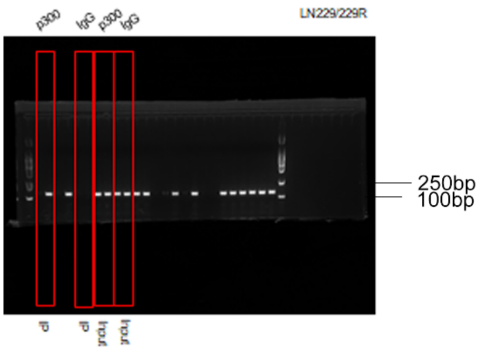

Figure 6B

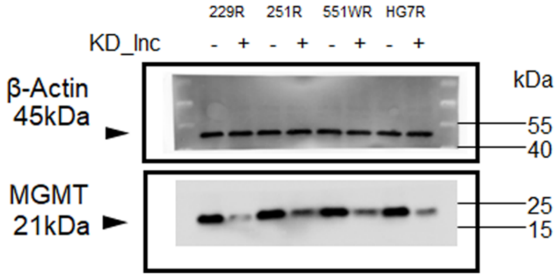

Figure 6E

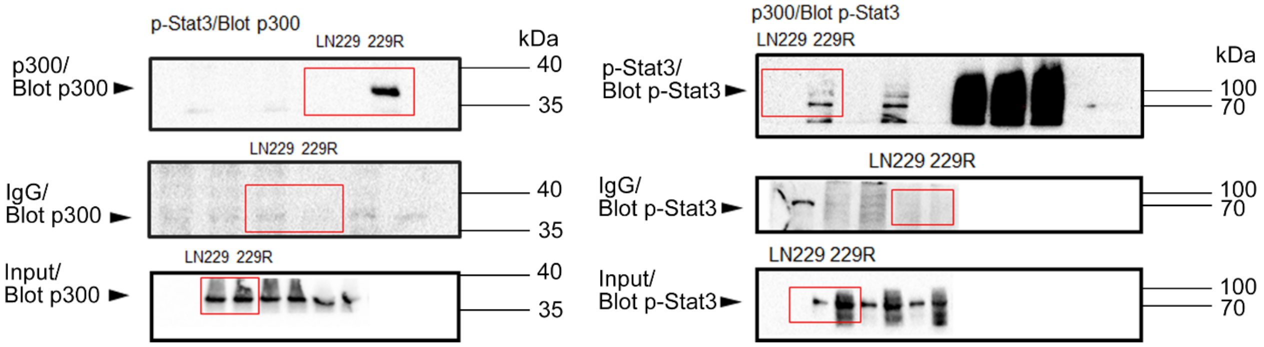

Figure 6F

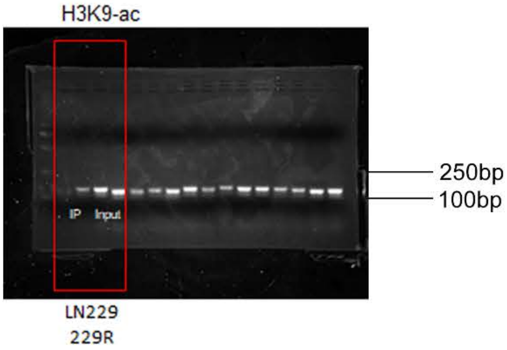

Figure 6G

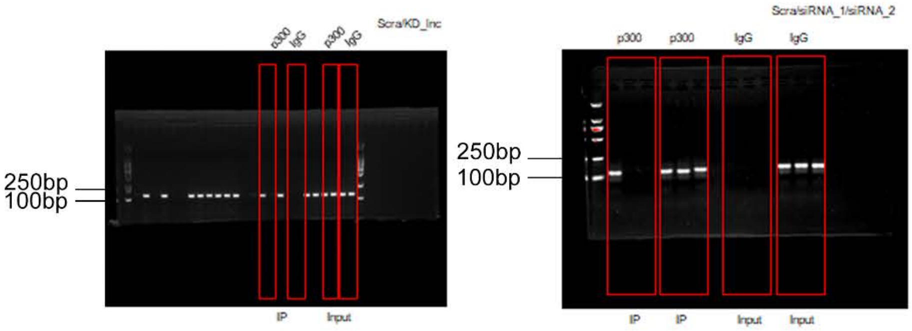

Figure 6H

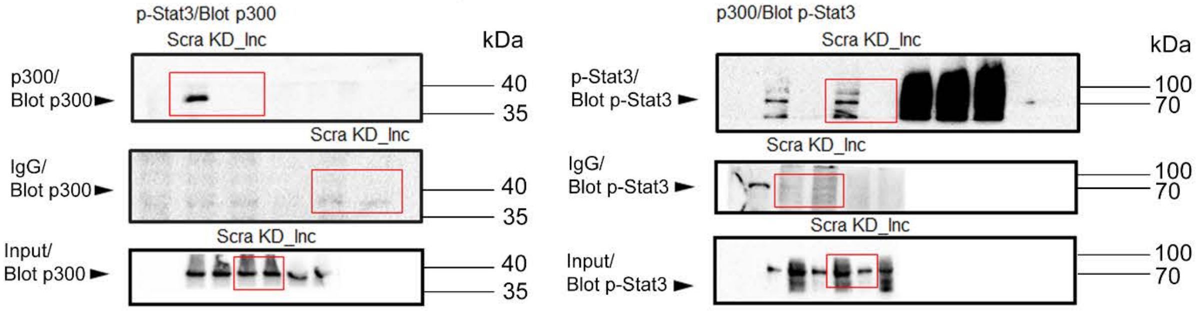

Figure 6I

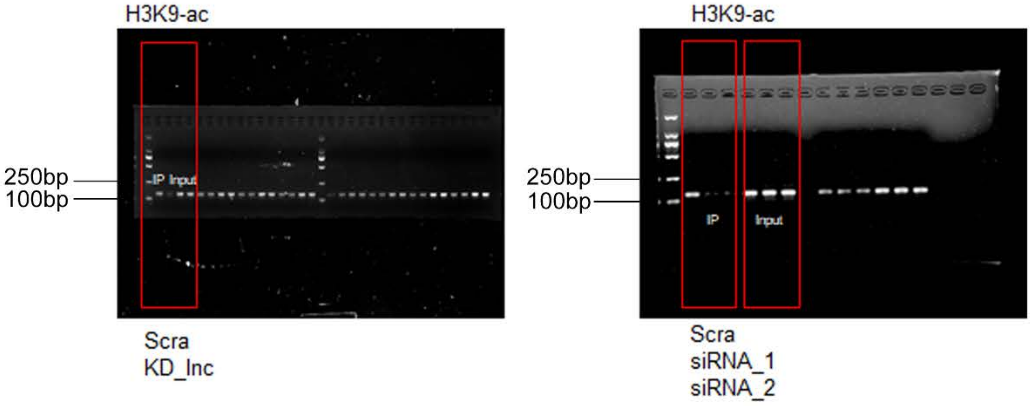

Figure 8E

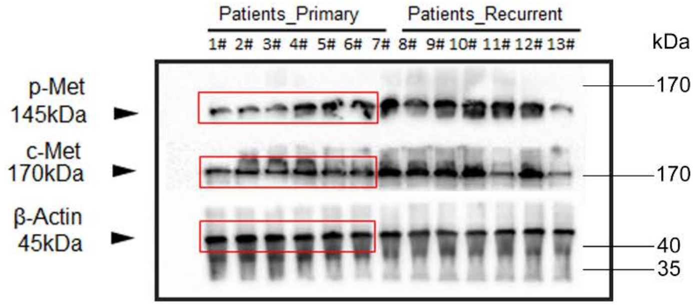

Supplementary Figure 5B

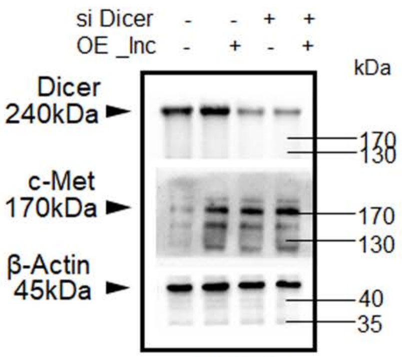

LN229

Supplementary Figure 5K

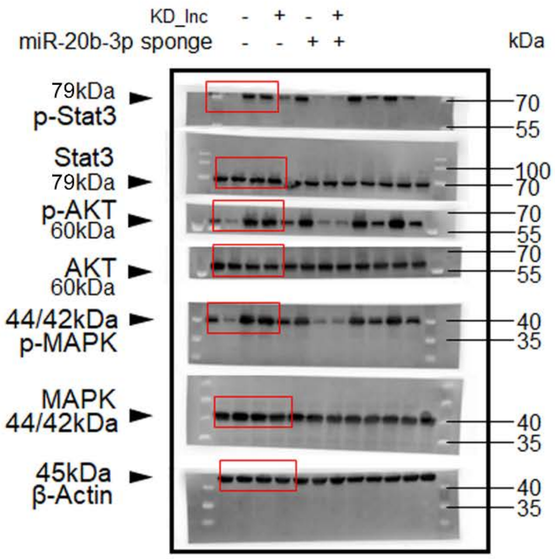

229R

Supplementary Figure 5L

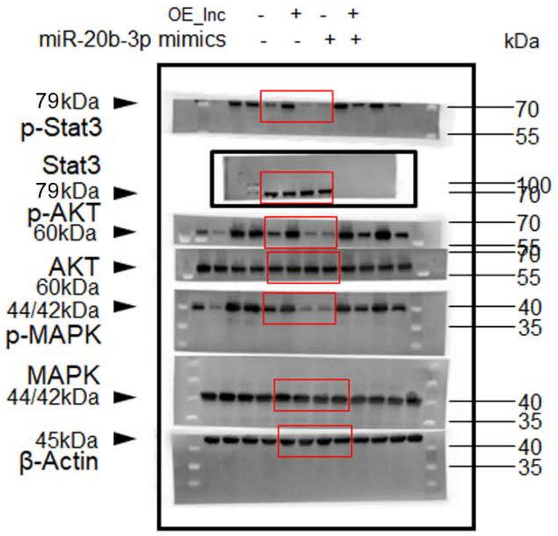

LN229

Supplementary Figure 6C

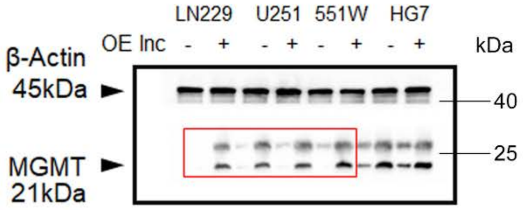

Supplementary Figure 6D

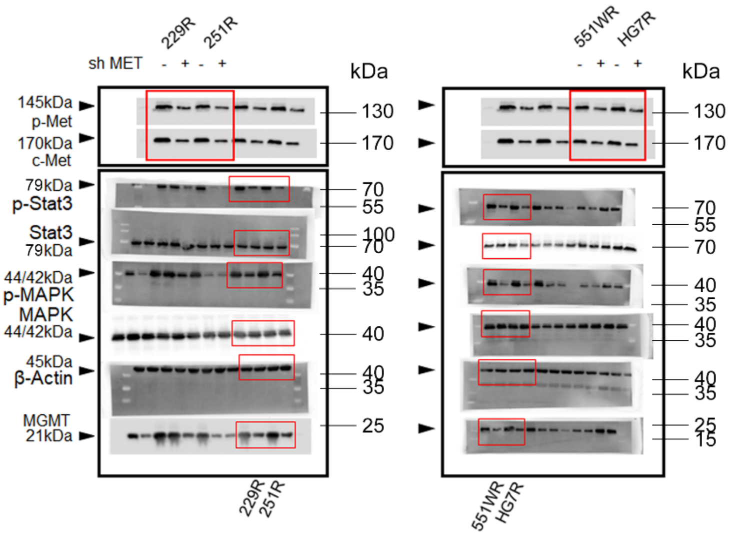

Supplementary Figure 6E

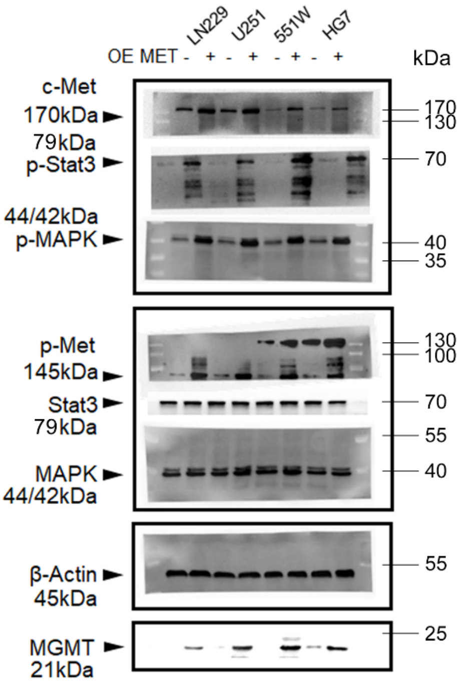

Supplementary Figure 6G

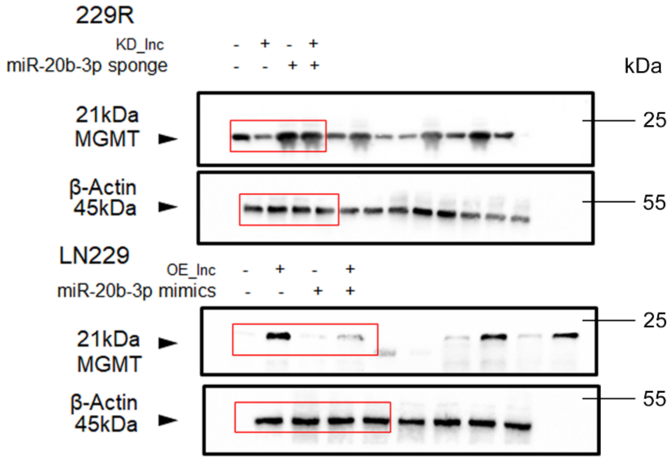

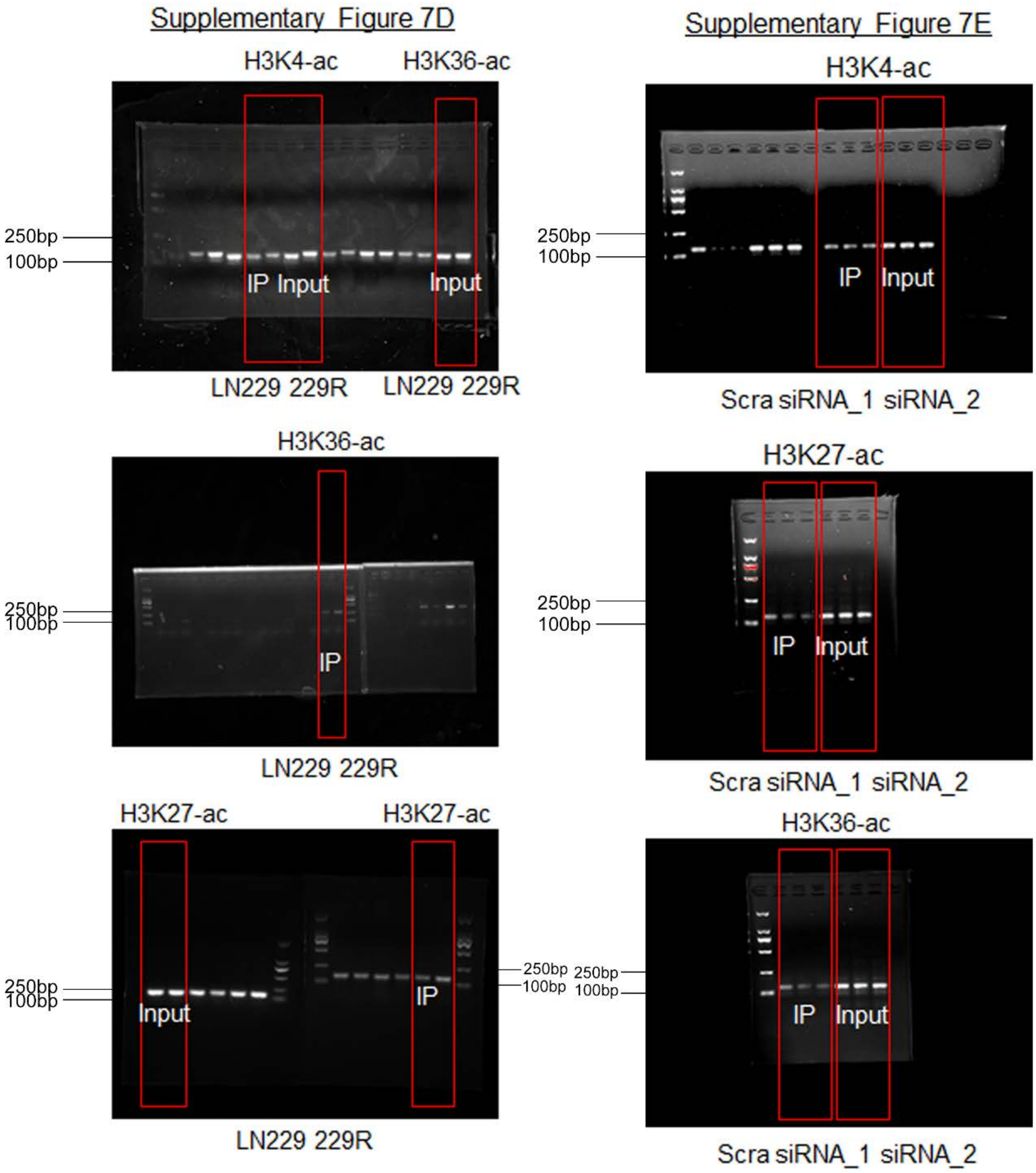

Supplementary Figure 9. Uncropped blots.

Uncropped images of all blots shown in main and supplementary figures. Molecular weight markers are indicated on the right side of each blot, whereas predicted molecular weight of the protein of interest indicated by an arrow on the left.

Supplementary Table 1. Predicted DNA binding elements of lnc-TALC gene for FOX families in the JASPAR database

Matrix ID	Name	Score	Relative score	Sequence ID	Start	End	Predicted sequence
MA0157.1	FOXO3	10.2323	0.935849513	hg38_refGene_NM_001282748	387	394	TCTAAACA
MA0157.1	FOXO3	10.2054	0.934945143	hg38_refGene_NM_001282748	832	839	GGAAAACA

Supplementary Table 2. Predicted miRNAs targeting to lnc-TALC and MET

DIANA & RegRNA2.0<br>17 common miRNAs targeting lnc-TALC	Targetscan & miRwalk & RegRNA & miRNA.org<br>72 common miRNAs targeting MET
hsa-miR-571	hsa-miR-335-3p
hsa-miR-3680-3p	hsa-miR-33a-3p
hsa-miR-1224-5p	hsa-miR-300
hsa-miR-1276	hsa-miR-205-3p
hsa-miR-142-3p	hsa-let-7f-1-3p
hsa-miR-302f	hsa-miR-361-5p
hsa-miR-3147	hsa-miR-206
hsa-miR-324-5p	hsa-let-7b-3p
hsa-miR-338-3p	hsa-miR-1284
hsa-miR-4259	hsa-miR-130a-3p
hsa-miR-4263	hsa-miR-34c-5p
hsa-miR-4310	hsa-miR-449a
hsa-miR-576-3p	hsa-miR-514b-5p
hsa-miR-657	hsa-miR-34b-3p
hsa-miR-20b-3p	hsa-miR-194-3p
hsa-miR-335-3p	hsa-miR-4307
hsa-miR-380-3p	hsa-miR-16-1-3p
	hsa-miR-195-3p
	hsa-miR-4302
	hsa-miR-16-2-3p
	hsa-miR-1205
	hsa-miR-4273
	hsa-miR-1912
	hsa-miR-1272
	hsa-miR-4255
	hsa-miR-337-3p
	hsa-miR-508-3p
	hsa-miR-198
	hsa-miR-1263
	hsa-miR-338-5p
	hsa-let-7f-2-3p
	hsa-miR-1262
	hsa-miR-183-3p
	hsa-miR-520h
	hsa-miR-2278
	hsa-miR-1267
	hsa-miR-30b-3p
	hsa-miR-4277
	hsa-miR-141-3p
	hsa-miR-3167
	hsa-miR-1204
	hsa-miR-2113
	hsa-miR-340-3p
	hsa-miR-148a-3p
	hsa-miR-3165
	hsa-miR-520d-5p
	hsa-miR-1290
	hsa-miR-485-5p
	hsa-miR-185-3p
	hsa-miR-1206
	hsa-miR-26b-3p
	hsa-miR-30a-3p
	hsa-miR-30e-3p
	hsa-miR-3138
	hsa-miR-1972
	hsa-miR-3143
	hsa-miR-2117
	hsa-miR-1293
	hsa-miR-342-5p
	hsa-miR-3128
	hsa-miR-15a-3p
	hsa-miR-3188
	hsa-miR-3130-3p
	hsa-miR-20b-3p
	hsa-miR-4303
	hsa-miR-509-3p
	hsa-miR-4327
	hsa-miR-30c-1-3p
	hsa-miR-30c-2-3p
	hsa-miR-3122
	hsa-miR-4283
	hsa-miR-4314

Supplementary Table 3. Clinical characteristics of primary and paired recurrent GBM patients

ID	Age	Gender	TMZ	Radiotherapy	KPS
#1613	48	F	1	1	100
#1405	54	M	1	1	80

Supplementary Table 4. Summary of clinical GBM patients

**Variables**		**Low lnc-TALC expression (n=38)**			**High lnc-TALC expression (n=39)**		
Temozolomide		Yes (n=22)	No (n=16)	P-value	Yes (n=18)	No (n=21)	P-value
Gender	Female	12	7	0.511	7	11	0.399
	Male	10	9		11	10	
Age	< 45	15	8	0.258	6	5	0.51
	⩾45	7	8		12	16	
KPS_Score	⩽70	6	4	1	2	1	0.889
	> 70	16	12		16	20	
Radiotherapy	Yes	11	9	0.703	18	0	0.001
	No	11	7		0	21	

Supplementary Table 5. Cox regression analysis of GBM with low lnc-TALC expression

Variables	Uni Cox regression			Mul Cox regression		
	HR	95%CI	p_value	HR	95%CI	p_value
Gender	0.750	0.297-1.890	0.542			
Age	3.778	1.355-10.528	0.011	2.545	0.903-7.172	0.077
KPS_Score	0.268	0.088-0.814	0.020	0.199	0.061-0.650	0.008
Temozolomid	0.268	0.095-0.756	0.013	0.287	0.095-0.866	0.027
Radiotherapy	0.639	0.249-1.637	0.351			

HR, hazard ratio; CI, confidence interval; KPS_Score, Karnofsky Performance Score (⩽70 vs. >70); Age (<45 vs. ⩾45 years); Gender (Female or Male); Temozolomide (Yes or No); Radiotherapy (Yes or No).

Supplementary Table 6. Top 10 up-regulated lncRNAs in TMZ-resistant GBM cells

Accession	Sequences
lnc-ERC1-1:5	CACCAAGAGCGAAACTCCATCTCAAAAAAAAAAAAAAAAAAATCAAACATAGAATTACCCTGTGGTCCAGCAATCCCACTTCTGGGCACATATGCAAAATTATTGACAGCAGGGTCTCAGAGAGAT<br>CCTTACACCCCTGTCCATAGCAGCCTCATTCACAGCAGCCAGAAGGTGGAACAACCCAAGTGTCCAGCTACAGATGAACGGACTTGTGAATTACGGCATAGACATACAAATGCTATTCCATCTCCAG<br>AGAGGGTTGAAGTTCCGACACACGCCACACAAAAATGAACCTCGAAGACATGCTGAGTGAAACAGGTCAGACACAAAAGGACAAATATTGTATGATCCCACTTAGACGAGCGACCTAGAACAGGC<br>AAATTCAGAGACGCAAAGGAGTCTTTCTGCCCATGTTCGCCTCATCTAACCCCTGCCTGCAGGGTCTGGGGGAGGTCAGAACGGGCAGTTAGGGTTGAAAGTATACAGAGTTTCAATCTGGGAAGAT<br>GAAAGAGTTCTGAGATGAAGGGTGGTGATGGTTGCAAACAGTGTAAATGTGTTTGATGCCACTGACCTGTACAGTTAAAAATGGCTACAGTGATAACTTCCGTGTTGTGCAAATTTTCACAATAAAA<br>ACTTAATGAAAGGTAGCTAGAGACAGCAAGTGAGGGGAAGCGAGGAAGGGCGAGTTATTGGTTGAAGGGTAGAGTTTTTGTTAGGGATGACGGGCGAAGTTTTGGGTACAGACTGTGGTCATGTTTC<br>CACAACATCGTGAATGAGTTTAGTGCCACAGCATTAAATGTATTCATGGTGTACTTGTGTACTTATGAATGGCTAAGATGATAAATATTATGTACATTTTACCACAATAAAAAAGTAATGAATTCTGG<br>CCAGGTACAGTGGCTCACCCCTGTAATCCCAGCACTTTGGGAGGCTAAGGCAGGCTGATCACTTGAGGTCAGGAATTCAAGACCAGCCCAGCCAACATGGCAAAACCCCATCTCTACTAAAAATAT<br>AGCCAGGCGTGGTGGCGCATACCTGTAATCTCTGCTATTTGGGAGACTGAGGCAGGAGAATCGCATGAACCCGGGAGGTGCAGGTTGCAGTGAGCCAAGATTGTACCACTGCACTCCAACCTGGGTT<br>ACAGCATCTC
lnc-TALC	TCACCCAGGTGCAAGCCCAGAGGCAGTCTATACCCCAACTCAACTGGCTGGTCCTCAATGCTGCCTGCTTCCGTGCCCAACTTAGAACTACACATCTGCTGCCTCTTGGTAACATGTCATTATAAGTC<br>TGAAGATTGCCATTTGAAATGCTCTTTGAGGGATGCGAAGTCAACCCTGGATCCAAAGTAGCTTTGATGTTTGTCAGGAAAATGCTGGAATTCTATACACTACAGTTTCTACAGAGCATGAAGAACTC<br>CAACTTCAGACAACCTGCAAAAAAAGTCAGAGAGCAATTAAATATAAAAATAAATTCCTTTGATAAAACAAA
lnc-RP11-15K19.2.1-3:6	GATAACGCCAAATGGTATATCACTGATTTTGTAGAGCTGCTGGGAGAACCGGAAGAATAACATCCATTGTCATACAGCTCCAAACAACTTCAGATGAATTTTTACAAGTTACACAGATTGATACTGT<br>TTGCTTACAATTGCCTATTACAACTTGCTATAGAAAGTTGGTACAGATGATCTGCACTGTCAAGTAAACTACAGTTAGGAATCCTCAAAGATTGGTTTGTTTGTTTTTAACTGTAGTTCCAGTATTATA<br>TGATCACTATCGATTTCCTGGAGAGTTTTGTAAT
lnc-SCG3-3:3	ACCATTGTGTATGATTCGTTGTTGACTGCAGCATCACTAGGTGGGACATCACTGCGTTTTCCTTGAACTTCCGCGGGTTCTCCTAAAGTTACTTGGTGACTTTTTATGATTGCTTTCTGCTCGTCTGAAT<br>AAAGGATGAGCCCAGGGCCTGCTGCCCACTGTCTGCTCTGATGACCCTCTCTCCCCTGCAGATCCGAGTGATGGTGGACCTGTGCAACAGCACCAAGGGCATCTGCCTCACAGGACCTTCTGGACCA<br>CCAGGACCTCCGGGAGCCGGCGGGTTGCCAGGACACAACGGATTGGATGGACAGCCTGGTCCTCAGGGCCCAAAAGGAGAAAAAGGAGCAAATGGAAAAAGAGGAAAAATGGGTATTTTTGGCAA<br>CTCTTCTAATTAATTTCCCTGTTATTTATCTCCATGATTGCATTTGGGTCGACTAAAACTGTGTGCAGCAGGGGTAAGGTGTTTCATCTCTGCACAGTTTAAGGCCGGTTTAACTCTTACCTAGAAGCC<br>ATTGCGTTCCGGGATACCAAGGGTATACTTCTTGGTCCCACCTTGACAGATGTTTATTTCAAACTGGAGAAGCCGTCTCCTGGGAACTGACTCGCACCCTTCTGCTGCAGATGGGAGGTGGAAGGAGC<br>AGGAGCCCACAGCCCTGGGAGGGGCATTCTGTGGGCCGTGTGACAGCTGGTGCTGGTGCATGCTCTTTCGAGGCTCAGCTCCTGCCTTGCCCTCTATTGCCTCCTCCTGCCCAGCGAATCTGTGTTTGG<br>TTGAAAATCAATGAACTGATGTTGGCCCCTGGTAAGGCATTTCTTGGCTAGAGGAATAGCTCAAACTGCTTGAGTGGAAAGGCAGCATCCTGGCCTCCCTGTGGCCGGCACTCAGGTCAAAGGGTCT<br>CCTCTTCAGGGGCTACAGGATAACTATGAAGATATGGCTGGCATCTCGGAGGAGCTTCCTTTGCCCTGTTATGAGCAGGCCATCACCACTGGAGAAAAGGCCCAGAAGCACCAAGGCACGAGATGT<br>GCCCAGGGAGCAGTAAGAATCAGGGGCTGGTGCAGGGCCTGGCAGGAGGCTGGGGCACAAAGGGGAAGATTCTGAAGGGCTCTGAGCACCAGTCCTGGGGAGCCGGGGTCAGGGCAGATCCACCT<br>GAAGAAAGCTGGCACATGCTGTGGTGGGAGCTCAAAGACAGAGGGTAAGAAACTACTCAGGAGGCTCCTACGAAGGCCTGGCTCTCAGCCACCCGTCAAAGCCACTGCTGAGGGCAGAGGGAGGT<br>TGTTTTCAGCCGGAGACTCCACTGCACCATCTTATTTTCAACCTCTTGGCTCTGCTACCCAAACTCACTTGGACTGGCCCTGGACGAGAAGAAATGGTAGAAAATTCTTCCAGACAGCAATTACTCCT<br>TTGGTCACCTCCACTCTTATTCTCATTAGCAATTGAAGTAAGGGCCAGTGGAGAGGGAAAAATTGTCGAGCACCTCTGACACTTGGCAGTGCCTACCTGCTTGTGTTCCAGAGGGCAGCTTCAGAGAA<br>AACGGGCACACACCGTCCAAGCCAGCTCAATCCTCAGAAGGCCAGTAATTACCATTGGAATTACACAGCAGCAATGGATCGTTCATATAGGATAATAATTATAATTATACATGAGCATCGCTGTAG<br>AATAATGATGCCTATTGGCTTATCCTGGGAAGACTCCAAAAATGTGATTCAGTCCTGAGGCCTCGGCAATGGGCATGATGAGGGCAGGCTCAGGGGCAGGAGGGCACCCTGCTAGAGCTGGGTGTGG<br>GGCACGTAGGAAAGGGGTGGTTTTCAAAAAGGCTTGAGACAGATCCCTGGCCTTGAGGATGTGCTTCGCATGCAGCCTGCAACCCAGGGAGTGCAGAAAAGAAAAAGTGCATTTGTTTATGGAGCA<br>CATATTTGTGTGGCAGGTACCATTCTAGATGCTGGAGATTCAGAAATAAGCCCCCCTCCTCCACCTTCACGAGGCTTACATCCTAGCTGGGAGAGACGGATAATAAATAAGATAAATAAATAG
NR_028415	AGGCTGCCTCCGGCTTCACCGCTCCTCCCCGAGCACAGCCTTTGTGGGGCCTACGGGGCAGTAGGAGACAGGACGCTTGTCCTGCGGAATGGCAAGGAATCAGCTCTCCATTTCAGGGCAAAGTGGG<br>GCCTGGATTGTGGGCCTTGAACATGAAGGCAGGTGGGCGAGGGACTAGGGGACCGCGTGAGGCCCGACGTGCACACGGCAGGGCCCGGCACCCATGGTTGCGTTCGGTCGGCCTTGGCCGGCTGGC<br>TCCTGCTTGGAAACTTGCTGGGTGGTGTCTGCGGGAAACAGTACTGAGTCTGGTGAGGAGGCAAACGCTCAGAATCTACTGCAGGTGTTAGTTCTGCAGAGGAGTCTTCCTGCTTAAGGGCTCATTTG<br>ATGTTCTGGAAATTTGCCAGGTACTGAAATACAGCCAGTCTTCCCAAAGATTCCTCCGCTCCTCTGAGTGCACGTGTGTGTGTGTGTGTGTGTGTGTGTACAGCCACACAACCTGACTCTTTGTTCACACA<br>CACGGGCCCTGGCCAAGTGTGAGGGTCTCCACGTGACGAAGGAAATGAGTAAACAACTTCAACATTGTTGTTTCCCTCTGAGGCTTGCAAAGAAGGTGTTAAGAGCACAAGATTTAAATCATAAACT<br>TTATTAAAAGCCAAACAGCCCAATGTGCGATTCTTGCCAACTTGGACAGAGTCTTTGGGGTAATGCTACACGGAGCTAATCCAGAAGGAATCGAGTGGAGGAAGACAGCAGGGTGGGGGTGAGCCC<br>ACGCGTCCAGCCAGGAGAGGCAGAGGGAGGCACAGCCCCGACACAGCCACCCACCCCGTGGCCAGAGCAGGGTGGCGCAGGAGCTGGCAAGACCTCTAAGCTGTTCCTGCTTCTGCTCTCCTCACA<br>CTGTTTCCTTAGCAAATGGAGCCTCCTTCACGCCTAAAGGGCCCAGAGCAAGGCAGGGGGAGGAGGGCCGACCCCTGCAAGACTGAGAACTGGCCAGAATTGCCCACTTGCTAGACTAGCAAGAGA<br>GCGAAGTCCCAGTGGGTACCCCCGACGAAGGACACCCGTTCCTTACCCCCCACCCAGAGGCACCCTTCCTCCTCCAAACACAAGATGCTGCTCCAGCAGGGGCAGGGACCATGTCAGCCTCCCACA<br>AGACACACACGGGAAAGCCCTGCTTGTGAGGGCGCCACACCCAGGGCAGGAATCAGAATGAAGACACGACTCCACAGTGAGCACGGGGCTCAGGACAGGACCCAGCGTGGCAGACCAGCCAGAG<br>AAGCTTTCACACTGCAAACAGCCCGCTCCACCTACACCCCGACTGGGCTCCACGACTCCCCGCAAGTGCCCTGCCACCTCAGGCACCTGCCCCCACGCCCACTCAGCGTCACCCCCTCGGCGGTTCA<br>TCTCCAGAAGGCTTGGAATTGATGACGTTTCCGCACCACGAGGGGCTTTTGCGATTCTGCGTCACTGAACTTGCATCTCATCCCCACGATCCGACATAAGCTTGCCAAGGACAGGACCTCGTGGCACC<br>CGGGACAGGGGCCACCCCATCCTACATGTGACAAAGCTGTCCCTCCGTGGAGCTGGCGTCTCTGATTTCCTCGGCCTGAGGGAGGCAGTGAAGATGAAGGAAGGAGGGAGGGCGCCTTGGTGGTGG<br>GTGCAGCTCTGTGCTGGTCTTTCTCTGGCCACCTAGACTTCAGTGGCCAAATGGCTTCAGGAGACCCACTGGTCTTTCCTGTTTTATTTTATTTTTTACTTTTTTTTTATTTTTATTTTTTGAGACAGAGTT<br>TCGCTCTTGGTGCCCAGGCTGAAGTGCAATGGTGCCATCTCAGCTCACCGCAACCTCTGCCTCCCAGGTTCAAGCAATTCTCCTGCCTCAGCCTCCCAAGTAGCTGAGATTACAGCCATGCACCACCA<br>CGCCTGGCTAATTTTTTGTATTTTTTTTTTTTTTTTAGTAGAGATAGGGATGAGCCACCGCGCCCAGCCAGTCTTTCCTGTATTAACAAAAGTATTGGTAGTAGCATTAGGTGTTAAAAATTATCAGCAC<br>AGCTTGGGACTGGCCACAAAATTCTTCTAAATCTCCCCAGGATAATCTCATGAGAGCACGGATGCTTTGCCCAGAATCTCTGCAACCGAGGAAGGTCTGTGGCACGGGGTGGGCCCTGAGAGACTTC<br>ACCTAAAAACTTTGGGTTCATTCCCCACCCGTGTGTGTATGTAGGTGTCTCTGTAGACAGTGATTAAAAATACTCCTGACT
NR_036444	AGCAGACAGCGCGGGCTGGGAGCGCCGGCAAGGGCGGCCCTCGCGCACCTGGCTGCCAGCCCGCAGGGGGCTCGCACGCAGACCTGCCCTTGCCACCTCAGCGCTCGGGTGCGGGCTGGGGGCGGT<br>GGAGCCACGAAAGGTGGTGCCGAGAGCGGAGCGCAGGACAGCGCAGGAGCCCCGGCGCATCGCGGCGCGCACAGACCCAGGATTTGTGAAGAGGTGACCCAAATTTCAAGACAATGTTGGATGCA<br>AACGGATTTGCCAGCAACTGCATCACTGTCAAAACCAGCTCTGCCCGTGTCTTCCGAAGATACCAAGTGAAATACATGTAGATGGGATTTACATCTGAAAAACCAAGTGAGAAACTGAAGACCAGA<br>GAGGGTGAGTGGTTTACTCAGTTTGGCACAACTGACCCCAACCTAGCCCTCCATGAGGACTGAGCGCATGAGAGATCCTGAGCCACAGCCGCCCAGCCCTGCTCCTCTCGAATTTCTGACCTACAGG<br>AACTGCAAGAAGTAATGAAAGACTGCTGTTTAAAGCCACTGCATTTTGGCATGATTTGTTATGCAGTCGTAGATAACCAGAAAACATCGGATTTACATTTTAAGGATTTGGTGCATGGGATGGGCTCA<br>GTTGGGCAGTTCTTCTGTTCCATGTGATGGTACTGGGGCTGCAGTCATTCAGAGGCTCAACTGGGCTGAACGTGCAAGGTTTCTTGCTCACGTGGCCATCAGTGGTGCTGGCTGCTGTTGGGAGCTCCA<br>CTGGACTTCTGTCCAGAGCTCCTCAGCTCACCTCTCTGTATCCTTGCCCTGTGGCTCAGGCTTCTAACAATACAGAGGCTGGATTCCAAGAGGAGGGAATTAGAAGCGTTCAGCCTTCTTAAGACCAG<br>GATTCAGATGTCCCAGACTGTCACTGTATTGACCGGAGCATTTACAGGCCAGCCTAGTAATTTTTCCATCTTGGGCAGGAATGGGTGGCATATGCTGGGGGTAGGGGTAGCATTGTTGGGGATCATCT<br>TTGGAGCTGTGTGCCAAGGAGTAAATTGCTGGGCTGCTTTCTACCTGAGGAAGTCCACTATGCTTGCTTGTGGAGAGTGGATTGTGTGCTCAGAGTCCACAAGGATTTAAGAATCCGAGCCCTGCTGC<br>CCTTGCCTCAGCCTGTGCATTAGGGCCCGGTGGTGAGAGCACTGAGCCATTGTGAATCAAGGCCCCTCCCTCGACCTGCATACTAACAGGCCAGAAGAGCTTGCTTGATTTTGGTCCTAGGCTCCTGC<br>CTGTGGGTTGGATTCCAGGGGCACTCTCTCCTCCACAAACCAGACGGTGGCAGGTACATCCCCTCAATGGAAATTGCCCACACTTCACCCTAAAGACAGATCCTGCTGTTCAGCAACAATGTGAGTG<br>GCCCACACATTTGTCCAGCATGAAACCGTTTGCAGATTCCTTTCCAGTACACAGTCCCGTCAGCCGGTCACTTCACGATGACAGCTCCTCGAAGCACCTTTTGGTTTGCAGTTGTGTAGCTTAGAAGT<br>AGGAGCCTGCACGGCTTCAGGAATTCACAGAAGGAGCACACTTTGTGCTTGGAGAAGTTAATTATAGGAATATCCTGCAACCTGCTGCTCTGAGCCTGTGCACCTGGGGTGTTTCCCTGGAGGTCCTG<br>TGTATGAGGATACTCATCAGCATGGGGTTTGGGAAGCCTAGCAGTGGAGACAACCTAGGTGGTCCTCTCCAGGGGAGTAGGTAAGAGCCCTACAGCAGTGAAAAGCATTGAAGGAAAAGTGTGTTC<br>AGCAGTGCTCTTAATCTGATCAGAGCAGCCTGAACACATTCTAAACACAGGGCATTTAGAACTAGGGTAGAGGCACTAGAGCACCATAGATACAAACTCAGATTCTGAAGGTAAATGCCCAGGTTC<br>AAATCCAAGCCCCCTCGCCAAGTGACCATGACTCCCCCGAGTCACCTGACCTCTGTGTTTACATCCTCAAAGTTAAGTAGTCAGGAAAGGGGGCTTGGGGCTTGCCACATGGATGGTTGCCACTCTC<br>CTTTGCAAAGTGTAGTCCCCAAAAACAGGTGGAAGAGGGAAGGGTCCAATTAGAAGGCCGAGTACCAGCTGCCATGAATATGCCAGAGCTGCTGGCTAGTGATAAATAACTGAAACCCAGGGGGA<br>CAGCAGAGCTTGGGTGACCATACATTCTAGGAAAGAAAACATGCCTGTTGTCCTGGCTTTTTATCCAGCTTAGACAAAGATGGGCAAAGAAAATAATTTAGAAAATGGTAGAGATGGGCACAGAGA<br>AAGGGCACGGAGGGAGGCACAAACAGAAGGCCAAGTGATGGTGGAGGCAGACATTGGAGAAATGCCTTTACAAGCCGGGGAATGCCAAGGATGCTGGCAACCCTGGGAGTGGGAAGAACCTGGA<br>ACACACCCTCCGTCAGAGTCTCCAGAAGGAACTGGCCCTTCATCACCTTTCGGACTTCTGGCCTCCAGAACTGGGCAAGAAAATGTTTCTGCTGTTGGAGGCCATCCAGCTGGTGGTCATTTGTTATG<br>GTTGCCACAGAAAACGAATATAGATTTAGACATCTAATTTGCATATATAGATATGCATGTGTATTTGTTAAATAAAAGATAAAGCATGACAACTCGATGGACAACGTTACAACTCATTAAATTGTGT<br>TGGTTAAAAAAAAAAAAAAAAAAAAAAA
ENST00000602425	TCAAATCCCTGCAGGGGGAGACCAGCAGCCCCGACTTCAGCCGCCATGCCTCCTGCCTGAGAAGCTAGCTGCGAAGGTTCCAGAAGGAGACTGCCCTCCGGGAACAGAAACAGCGCATGACTGCGA<br>CCTGTCTGCAAAAATCTTGATCACTTGTAAATCCTTCTCCACCTGATAAGTTATCACATTGGCTCATGATGTTTTTTTCATTCTCACCCTATGGCGTGGATGCTGTTACTACCCCATTTACAAAGGAG<br>TAAAGTGAGGCCCAGAGAGAACAGTTAAATCACTTCCCCAGCTTCACTCAACTCATCTACGGTGAGTCGACGGTACACATGCCCTTCCACAACACAAAGGTCTCCACTTTGCCACGTGTCACTCTGA<br>TGGTTCCAGGTGCAGATTTTGCGTCTGCTTCTTGACTTCTAATGGCTGCACCATTTTGCATTGGATGGTTATACCATCATTTATTTGACTCATCTCCTGTGCACAAGCATCTAGTTTATCTGCAAACTTTT<br>GCTACCAAAAACAATGCTGCAATGAATAAATAAGCTGATGCCTGCTTCTA
NONHSAT025399	AGGCTGCCTCCGGCTTCACCGCTCCTCCCCGAGCACAGCCTTTGTGGGGCCTACGGGGCAGTAGGAGACAGGACGCTTGTCCTGCGGAATGGCAAGGAATCAGCTCTCCATTTCAGGGCAAAGTGGG<br>GCCTGGATTGTGGGCCTTGAACATGAAGGCAGGTGGGCGAGGGACTAGGGGACCGCGTGAGGCCCGACGTGCACACGGCAGGGCCCGGCACCCATGGTTGCGTTCGGTCGGCCTTGGCCGGCTGGC<br>TCCTGCTTGGAAACTTGCTGGGTGGTGTCTGCGGGAAACAGTACTGAGTCTGGTGAGGAGGCAAACGCTCAGAATCTACTGCAGGTGTTAGTTCTGCAGAGGAGTCTTCCTGCTTAAGGGCTCATTTG<br>ATGTTCTGGAAATTAGCTGGCGTCTCTGATTTCCTCGGCCTGAGGGAGGCAGTGAAGATGAAGGAAGGAGGGAGGGCGCCTTGGTGGTGGGTGCAGCTCTGTGCTGGTCTTTCTCTGGCCACCTAGAC<br>TTCAGTGGCCAAATGGCTTCAGGAGACCCACTGGTCTTTCCTGTTTTATTTTATTTTTTACTTTTTTTTTATTTTTATTTTTTGAGACAGAGTTTCGCTCTTGGTGCCCAGGCTGAAGTGCAATGGTGCCA<br>TCTCAGCTCACCGCAACCTCTGCCTCCCAGGTTCAAGCAATTCTCCTGCCTCAGCCTCCCAAGTAGCTGAGATTACAGCCATGCACCACCACGCCTGGCTAATTTTTTGTATTTTTTTTTTTTTTTTAGT<br>AGAGATAGGGATGAGCCACCGCGCCCAGCCAGTCTTTCCTGTATTAACAAAAGTATTGGTAGTAGCATTAGGTGTTAAAAATTATCAGCACAGCTTGGGACTGGCCACAAAATTCTTCTAAATCTCC<br>CCAGGATAATCTCATGAGAGCACGGATGCTTTGCCCAGAATCTCTGCAACCGAGGAAGGTCTGTGGCACGGGGTGGGCCCTGAGAGACTTCACCTAAAAACTTTGGGTTCATTCCCCACCCGTGTGT<br>GTATGTAGGTGTCTCTGTAGACAGTGATTAAAAATACTCCTGACT
ENST00000549807	CCATCTTTTCTCTAAGGTCTTCAACTAATTGGATGAAGCCCATCCACATTATGGAGGGTCACTAGCTTTACTCAAAGCCTACTGATTTCATTGTAAGTCACATCTAAAAAATACCTTCACAGCAACAT<br>CTATGCTTTTATCTGCCAAACAACTGGGCACCGTATGCTAGCCAAGTTGACACATAAAATTAACTGTCACTCCATGGGACTAGATGAAATTACCCAGGAGTTAATGTATTTAGAGAAATAAAGATGG<br>CCCAGGACAGAGCCCTGGCACGCTCAAACTGCAAAAGGGGAAAAGAACCCAGCAATGGAGCCTAAGAAGGAGCTGGCAGGAGGTAGGAGGAAAACCAAGACTGTGTGGTGTCTCAGAAGCCAAT<br>GAAGAAGTGCTCCAAAAAGGAAGATCAACTATTTCAAGCACTGCCAAGAAATCCTATAAGGCCCTGGGAAGATTACGTAGCCAACACTGGTGTGAAAATCATGCCTATGGAGGGTTCTTTTGGAAC<br>CCAGAAGAAACAGATAAAGGAGGTGTTTATTCATGAAACCAGCACTTAGAAGACTGCATCAGCAGTTCCAGCTCCATGATTACAAGCTCCTCGAAGACATGAACCAGATCACACCTCTCCTGTGTG<br>GCTAAGGCCAACTGCACATGTAGAACGGTGTTCCTCCTATGCTTGGGACAAATAAATCTCACAAAATCAA
lnc-HIST1H2BI-1:1	AAACATTTTCTCATTAGTCCTGTTTGCCTAGTCAGTCACTTGTATAGACAGGGCAACAGCACGGCCTGAATATTGGGCAGGACACCGCCCTGCGCGATAGTAACTTTGCCCAACAGCTTGTTGAGCTT<br>CTCGTCGTGGATGGCGAGCTGCAGGCGTCGGGGGGTGACGCGGGTCTTCTTGTCTCGGGCCGCGTTGCCCGCCAACTCCAGGATCTTGGCGGTCAGGTACTCCAGCACCGCCTCCAGGTACACCGGC<br>GCACCGGCCCGGACCCGCTTGGCGTAGTTGCCCTTGCGGAGGAGGCGGTGCACTCGGCCCACAGAAAACTGGAGACCAGCCCTGGAAGAGTGCGTTTTAGCCTTGGCGCGAGCTTTGCTGCCCTGCA<br>TTCCACATCCAGACGCAGTGAAGTATTTAAATACTTGTTTAGAAGAAGGAAAGTGATTGATTCTAGTAAAACGCAAGAGCTAAACTCTTATAATCTATATTCGGCAAAGTAGACTGTGCTATCTCCTG<br>AACACAGATTTCAGCCAATCGAAAAGCAGGTGTTTAAAAAAATTT

Supplementary Table 7. The gene-specific primers used for the nested-PCR of the RACE analysis on lnc-TALC

Gene specific primer	Sequence
3' RACE GSP1	CCAACTCAACTGGCTGGTCCTCAATG
3' RACE GSP2	TGGTAACATGTCATTATAAGTCTGAAGAT
5' RACE GSP1	GCTCTCTGACTTTTTTTGCAGGTTGTCTG
5' RACE GSP2	CTTCATGCTCTGTAGAAACTGTAGTGTAT

Supplementary Table 8. Sequences of siRNA against specific targets

Dicer siRNA	Sense (5’-3’)	GGGCACCCAUCUCUAAUUA	
	Anti-sense (5’-3’)	UAAUUAGAGAUGGGUGCCC	
AKT siRNA	Sense (5’-3’)	CCUGCCCUUCUACAACCAGGA	UACUCAAACUCGUUCAUGGUC
	Anti-sense (5’-3’)	UCCUGGUUGUAGAAGGGCAGG	CCAUGAACGAGUUUGAGUACC
MGMT siRNA	Sense (5’-3’)	UUUCAUUUCACAAUCCUUGUC	
	Anti-sense (5’-3’)	CAAGGAUUGUGAAAUGAAACG	
Stat3 siRNA	Sense (5’-3’)	AGAAAUGAAGGCAAAACGGGG	UUAUGAAACACCAAAGUGGCA
	Anti-sense (5’-3’)	CCGUUUUGCCUUCAUUUCUAA	CCACUUUGGUGUUUCAUAAUC
MAPK siRNA	Sense (5’-3’)	UGGUUUUUGGGGAAAUACCAG	
	Anti-sense (5’-3’)	GGUAUUUCCCCAAAAACCACC	
lnc-TALC siRNA	Sense (5’-3’)	AUAAUGACAUGUUACCAAGAG	UUCAAAUGGCAAUCUUCAGAC
	Anti-sense (5’-3’)	CUUGGUAACAUGUCAUUAUAA	CUGAAGAUUGCCAUUUGAAAU
lnc-ERC1-1:5 siRNA	Sense (5’-3’)	UUUUGAGAUGGAGUUUCGCUC	UUUUUUUUUUUUUGAGAUGGA
	Anti-sense (5’-3’)	GCGAAACUCCAUCUCAAAAAA	CAUCUCAAAAAAAAAAAAAAA
lnc-RP11-15K19.2.1-3:6 siRNA	Sense (5’-3’)	ACAAAAUCAGUGAUAUACCAU	AUGACAAUGGAUGUUAUUCUU
	Anti-sense (5’-3’)	GGUAUAUCACUGAUUUUGUAG	GAAUAACAUCCAUUGUCAUAC
lnc-SCG3-3:3 siRNA	Sense (5’-3’)	UAAAAAGUCACCAAGUAACUU	UCCUUUUUCUCCUUUUGGGCC
	Anti-sense (5’-3’)	GUUACUUGGUGACUUUUUAUG	CCCAAAAGGAGAAAAAGGAGC
NR_028415 siRNA	Sense (5’-3’)	AAUUUUUAACACCUAAUGCUA	UGUUCAAGGCCCACAAUCCAG
	Anti-sense (5’-3’)	GCAUUAGGUGUUAAAAAUUAU	GGAUUGUGGGCCUUGAACAUG
NR_036444 siRNA	Sense (5’-3’)	ACAUUGUCUUGAAAUUUGGGU	AUUUCACUUGGUAUCUUCGGA
	Anti-sense (5’-3’)	CCAAAUUUCAAGACAAUGUUG	CGAAGAUACCAAGUGAAAUAC
ENST00000602425 siRNA	Sense (5’-3’)	AAUGUGAUAACUUAUCAGGUG	UAAAAUGGGGUAGUAACAGCA
	Anti-sense (5’-3’)	CCUGAUAAGUUAUCACAUUGG	CUGUUACUACCCCAUUUUACA
NONHSAT025399 siRNA	Sense (5’-3’)	UGUUCAAGGCCCACAAUCCAG	AUCAAAUGAGCCCUUAAGCAG
	Anti-sense (5’-3’)	GGAUUGUGGGCCUUGAACAUG	GCUUAAGGGCUCAUUUGAUGU
ENST00000549807 siRNA	Sense (5’-3’)	UGACUUACAAUGAAAUCAGUA	AAGGUAUUUUUUAGAUGUGAC
	Anti-sense (5’-3’)	CUGAUUUCAUUGUAAGUCACA	CACAUCUAAAAAAUACCUUCA
lnc-HIST1H2BI-1:1 siRNA	Sense (5’-3’)	ACAAGUAUUUAAAUACUUCAC	UAGAUUAUAAGAGUUUAGCUC
	Anti-sense (5’-3’)	GAAGUAUUUAAAUACUUGUUU	GCUAAACUCUUAUAAUCUAUA

Supplementary Table 9. Sequences of sgRNA for knockdown of lnc-TALC

sgRNA1	TAGCATGCATAGCATAAATC
sgRNA2	AATGGATTTCCAACGAGTTC
sgRNA3	ATCCACATTCCAGAACTCGT
sgRNA4	ATATTGCACTTAAGGATAGG

Supplementary Table 10. Primary antibodies used in the present study

Antigens	Manufacturer	Catalogue numbers	Application
c-Met	Cell Signaling Technology	#8198	1:1000 for WB, 1:100 for IHC
p-Met(Tyr1234/1235)	Cell Signaling Technology	#3077	1:1000 for WB
AKT	Cell Signaling Technology	#4685	1:1000 for WB
p-AKT(S473)	Cell Signaling Technology	#4060	1:1000 for WB, 1:200 for IHC
MAPK	Cell Signaling Technology	#4695	1:1000 for WB
p-MAPK(Thr 202/Tyr 20	Cell Signaling Technology	#4370	1:1000 for WB, 1:200 for IHC
STAT3	Cell Signaling Technology	#9139	1:1000 for WB
p-STAT3(Y705)	Cell Signaling Technology	#9145	1:1000 for WB, 1:200 for IHC, 1:50 for IP
MGMT	Abcam	ab39253	1:500 for WB, 1:100 for IF, 1:50for IHC
FOXO3	Abcam	ab12162	1:1000 for WB, 1:200 for IF,1:50 for ChIP
Ago2	Abcam	ab32381	1:50 for RIP
Dicer	Cell Signaling Technology	#5362	1:1000 for WB
β-Actin	Zsbio Store	PR-0255	1:1000 for WB
H3K4 (ac)	Abcam	ab176799	1:50 for ChIP
H3K9 (ac)	Abcam	ab4441	1:50 for ChIP
H3K27 (ac)	Abcam	ab4729	1:50 for ChIP
H3K36 (ac)	Abcam	ab177179	1:50 for ChIP
p300	Abcam	ab14984	1:50 for ChIP, 1:50 for IP

Supplementary Table 11. Sequences of primers used for qRT-PCR

lnc-TALC	Forward (5'-3')	CTGGTCCTCAATGCTGCCT
	Reverse (5'-3')	ATCCAGGGTTGACTTCGCAT
MET	Forward (5'-3')	AGTGAGAAGGCTAAAGGAAACG
	Reverse (5'-3')	TTTGGACCGTCAAGAAGTAAAT
MGMT	Forward (5'-3')	AGGAGCGATGAGGAGCAATC
	Reverse (5'-3')	GGCACCGTCACTGCGAAT
β-Actin	Forward (5'-3')	AATCGTGCGTGACATTAAGGAG
	Reverse (5'-3')	ACTGTGTTGGCGTACAGGTCTT
lnc-ERC1-1:5	Forward (5'-3')	CCCAAGTGTCCAGCTACAGA
	Reverse (5'-3')	TCGCTCGTCTAAGTGGGATC
lnc-RP11-15K19.2.1	Forward (5'-3')	ACAGCTCCAAACAACTTCAGA
	Reverse (5'-3')	ACAAAACTCTCCAGGAAATCGA
lnc-SCG3-3:3	Forward (5'-3')	TTATGAGCAGGCCATCACCA
	Reverse (5'-3')	ACCCTCTGTCTTTGAGCTCC
NR_028415	Forward (5'-3')	GCGATTCTGCGTCACTGAAC
	Reverse (5'-3')	CCTCAGGCCGAGGAAATCAG
NR_036444	Forward (5'-3')	TAGAAGTAGGAGCCTGCACG
	Reverse (5'-3')	ACCTAGGTTGTCTCCACTGC
ENST00000602425	Forward (5'-3')	TTCCCAAAGATTCCTCCGCT
	Reverse (5'-3')	AATCGCACATTGGGCTGTTT
NONHSAT025399	Forward (5'-3')	GGAGGCAAACGCTCAGAATC
	Reverse (5'-3')	TGGCCACTGAAGTCTAGGTG
ENST00000549807	Forward (5'-3')	GCTCAAACTGCAAAAGGGGA
	Reverse (5'-3')	TACGTAATCTTCCCAGGGCC
lnc-HIST1H2BI-1:1	Forward (5'-3')	AACTTTGCCCAACAGCTTGT
	Reverse (5'-3')	GTTTTCTGTGGGCCGAGTG
U6	Forward (5'-3')	CTCGCTTCGGCAGCACA
	Reverse (5'-3')	AACGCTTCACGAATTTGCGT
miRNA-648	Forward (5'-3')	CACAGACACCTCCAAGTG
miRNA-767-3p	Forward (5'-3')	CCATGGTTGTCTGAGCA
miRNA-221	Forward (5'-3')	GTCCAGTTTTTTTTTTTTTTTGAAAC
miRNA-181b	Forward (5'-3')	ACACTCCAGCTGGGAACATTCATTGCTGTCGG
miRNA-181d	Forward (5'-3')	CTGGAGAACATTCATTGTTGTC
miRNA-20b-3p	Forward (5'-3')	ATGCCAAAGTGCTCATAGT
miRNA-335-3p	Forward (5'-3')	TTTTTCATTATTGCTCCTGACC

Supplementary Table 12. Sequences of predicted and mutant binding sites used for luciferase reporter assay

Name	Sequence
MET-3'URT (miRNA-20b-3p)	AGACAGGATCTCACTCTGTTGCCAGGGCTGTAGTGCAGTGGTGTGATCATAGCTCAC TGCAACCTCCACCTCCCAGGCTCAAGCCTCCCGAATAGCTGGGACTACAGGCGCACA CCACCATCCCCGGCTAATTTTTGTATTTTTTGTAGAGACGGGGTTTTGCCATGTTGCC AAGGCTGGTTTCAAACTCCTGGACTCAAGAAATCC
MET-3'URT-Mut (miRNA-20b-3p)	AGACAGGATCTCACTCTGTTGCCAGGGCTGTAGTGCAGTGGTGTGATCATAGCTCAC TGCAACCTCCACCTCCCAGGCTCAAGCCTCCCGAATAGCTGGGCAGCACTGCGCACA CCACCATCCCCGGCTAATTTTTGTATTTTTTGTAGAGACGGGGTTTTGCCATGTTGCC AAGGCTGGTTTCAAACTCCTGGACTCAAGAAATCC
lnc-TALC (miRNA-20b-3p)	TCTGAAGATTGCCATTTGAAATGCTCTTTGAGGGATGCGAAGTCAACCCTGGATCCA AAGTAGCTTTGATGTTTGTCAGGAAAATGCTGGAATTCTATACACTACAGTTTCTACA GAGCATGAAGAACTCCAACTTCAGACAACCTGCAAAAAAAGTCAGAGAGCAATTAA ATATAAAAATAAATTCCTTTGATAAAACAAA
lnc-TALC-Mut (miRNA-20b-3p)	TCTGAAGATTGCCATTTGAAATGCTCTTTGAGGGATGCGAAGTCAACCCTGGATCCA AAGTAGCTTTGATGTTTGTCAGGAAAATGCTGGAATTCTATACCAGCACTTTTAGCA CTAGCATGAAGAACTCCAACTTCAGACAACCTGCAAAAAAAGTCAGAGAGCAATTA AATATAAAAATAAATTCCTTTGATAAAACAAA
MET-3'URT (miRNA-335-3p)	AGACATCATAGTGCTAGTACTATGTCAAAGCAACAGTCCACACTTTGTCCAATGGTT TTTTCACTGCCTGACCTTTAAAAGGCCATCGATATTCTTTGCTCTTGCCAAAATTGCA CTATTATAGGACTTGTATTGTTATTTAAATTACTGGATTCTAAGGAATTTCTTATCTG ACAGAGCATCAGAACCAGAGGCTTGGTCCCACA
MET-3'URT-Mut (miRNA-335-3p)	AGACATCATAGTGCTAGTACTATGTCAAAGCAACAGTCCACACTTTGTCCAATGGTT TTTTCACTGCCTGACCTTTAAAAGGCCATCGATATTCTTTTAGAGGTCCAAAATTGCA CTATTATAGGACTTGTATTGTTATTTAAATTACTGGATTCTAAGGAATTTCTTATCTG ACAGAGCATCAGAACCAGAGGCTTGGTCCCACA
lnc-TALC (miRNA-335-3p)	TCTGAAGATTGCCATTTGAAATGCTCTTTGAGGGATGCGAAGTCAACCCTGGATCCA AAGTAGCTTTGATGTTTGTCAGGAAAATGCTGGAATTCTATACACTACAGTTTCTACA GAGCATGAAGAACTCCAACTTCAGACAACCTGCAAAAAAAGTCAGAGAGCAATTAA ATATAAAAATAAATTCCTTTGATAAAACAAA
lnc-TALC-Mut (miRNA-335-3p)	TCTGAAGATTGCCATTTGAAATGCTCTTTGAGGGATGCGAAGTCAACCCTGGATCCA AAGTAGCTTTGATGTTTGTCAGGAAAATGCTGGAATTCTATACACTACAGTTTCTACA GAGCATGAAGAACTCCAACTTCAGACAACCTGCAAAAAAAGGACTATCTACCGTAA ATCGACCCCTAAATTCCTTTGATAAAACAAA

Supplementary Table 13. Probes sequence for lnc-TALC

5'-TTTTTTGCAGGTTGTCTGAAGTTGGAGTTCTTCATGCTCTGTAGAAACTGTAGTGTATAG-3'

Supplementary Table 14. Sequences of primers used for ChIP-PCR

Primer	Sequence
MGMT_primer	Forward 5'GCAGCCTGTATTGTCACCAG 3'
	Reverse 5'CGGTGAAGTTTCCCTGTTGG 3'
lnc-TALC_primer 1	Forward 5'AATTAGTCCGCATTCCTGGC 3'
	Reverse 5'TGGCGGCAAAATAAAGAGGG 3'
lnc-TALC_primer 2	Forward 5'GCTAAGGAACGTCAGCCAAG 3'
	Reverse 5'TGCCCATCATTTCACTGCTG 3'
lnc-TALC_primer 3	Forward 5'AGATCGTTGCTGTGAAGTGAAAA 3'
	Reverse 5'CTCTGCGTCCCACTCCAAA 3'
