# Supplementary material for: Lnc-TALC promotes O6-methylguanine-DNA methyltransferase expression via regulating the c-Met pathway by competitively binding with miR-20b-3p
Source: Nat Commun. 2019 May 3;10:2045. doi: 10.1038/s41467-019-10025-2 (PMC6499807; doi:10.1038/s41467-019-10025-2)
Supplement: Supplementary file 3 — Reporting Summary [file 41467_2019_10025_MOESM3_ESM.pdf]

nature research

Corresponding author(s): Jinquan Cai, Chunsheng Kang, Chuanlu Jiang

Last updated by author(s): Mar 10, 2019

Reporting Summary

Nature Research wishes to improve the reproducibility of the work that we publish. This form provides structure for consistency and transparency in reporting. For further information on Nature Research policies, see Authors & Referees and the Editorial Policy Checklist.

Statistics

For all statistical analyses, confirm that the following items are present in the figure legend, table legend, main text, or Methods section.

n/a	Confirmed	
☐	☒	The exact sample size (*n*) for each experimental group/condition, given as a discrete number and unit of measurement
☐	☒	A statement on whether measurements were taken from distinct samples or whether the same sample was measured repeatedly
☐	☒	The statistical test(s) used AND whether they are one- or two-sided<br>*Only common tests should be described solely by name; describe more complex techniques in the Methods section.*
☐	☒	A description of all covariates tested
☐	☒	A description of any assumptions or corrections, such as tests of normality and adjustment for multiple comparisons
☐	☒	A full description of the statistical parameters including central tendency (e.g. means) or other basic estimates (e.g. regression coefficient) AND variation (e.g. standard deviation) or associated estimates of uncertainty (e.g. confidence intervals)
☐	☒	For null hypothesis testing, the test statistic (e.g. *F*, *t*, *r*) with confidence intervals, effect sizes, degrees of freedom and *P* value noted<br>*Give P values as exact values whenever suitable.*
☒	☐	For Bayesian analysis, information on the choice of priors and Markov chain Monte Carlo settings
☒	☐	For hierarchical and complex designs, identification of the appropriate level for tests and full reporting of outcomes
☐	☒	Estimates of effect sizes (e.g. Cohen's *d*, Pearson's *r*), indicating how they were calculated

Our web collection on statistics for biologists contains articles on many of the points above.

Software and code

Policy information about availability of computer code

Data collection	No software was used for data collection.
Data analysis	GraphPad software version 7.0 (GraphPad Software, CA, USA); IBM SPSS Statistics 23.0 (SPSS, Chicago, USA); Gene Cluster 3.0 and Gene Tree View; R version 3.5.1 (Feather Spray) and Flowjo 10 were used for data analysis and visulization.

For manuscripts utilizing custom algorithms or software that are central to the research but not yet described in published literature, software must be made available to editors/reviewers. We strongly encourage code deposition in a community repository (e.g. GitHub). See the Nature Research guidelines for submitting code & software for further information.

Data

Policy information about availability of data

All manuscripts must include a data availability statement. This statement should provide the following information, where applicable:

- Accession codes, unique identifiers, or web links for publicly available datasets
- A list of figures that have associated raw data
- A description of any restrictions on data availability

Source data in this study are available on https://www.ncbi.nlm.nih.gov/geo/query/acc.cgi?acc=GSE113510, https://www.ncbi.nlm.nih.gov/geo/query/acc.cgi?acc=GSE32466, http://www.cgga.org.cn, http://cancergenome.nih.gov/, The authors declare that all data supporting the findings of this study are available within the paper and the Supplementary Material.

Field-specific reporting

Please select the one below that is the best fit for your research. If you are not sure, read the appropriate sections before making your selection.

☒ Life sciences ☐ Behavioural & social sciences ☐ Ecological, evolutionary & environmental sciences

For a reference copy of the document with all sections, see nature.com/documents/nr-reporting-summary-flat.pdf

Life sciences study design

All studies must disclose on these points even when the disclosure is negative.

Sample size	The choice of sample size has been reported in a large amount of literature.
Data exclusions	No data were excluded from the analyses.
Replication	All experiments were successfully repeated st least 3 times.
Randomization	No randomization was performed.
Blinding	No blinding was performed in this study.

Reporting for specific materials, systems and methods

We require information from authors about some types of materials, experimental systems and methods used in many studies. Here, indicate whether each material, system or method listed is relevant to your study. If you are not sure if a list item applies to your research, read the appropriate section before selecting a response.

Materials & experimental systems

n/a	Involved in the study	
☐	☒	Antibodies
☐	☒	Eukaryotic cell lines
☒	☐	Palaeontology
☐	☒	Animals and other organisms
☒	☐	Human research participants
☒	☐	Clinical data

Methods

n/a	Involved in the study	
☒	☐	ChIP-seq
☐	☒	Flow cytometry
☒	☐	MRI-based neuroimaging

Antibodies

Antibodies used	c-Met Rabbit mAb #8198; p-Met(Tyr1234/1235)Rabbit mAb #3077; AKT Rabbit mAb #4685; Phospho-Akt (Ser473) Rabbit mAb #4060; p44/42 MAPK (Erk1/2) Rabbit mAb #4695; Stat3 (124H6) Mouse mAb #9139; Phospho-Stat3 (Tyr705) Rabbit mAb #9145; Anti-MGMT antibody [MT3.1] (ab39253); Anti-FOXO3A antibody - ChIP Grade (ab12162); Anti-Argonaute-2 antibody - ChIP Grade (ab32381); Dicer (D38E7) Rabbit mAb #5362; Anti-Histone H3 (acetyl K4) antibody [EPR16596] - ChIP Grade (ab176799); Anti-Histone H3 (acetyl K9) antibody - ChIP Grade (ab4441); Anti-Histone H3 (acetyl K27) antibody - ChIP Grade (ab4729); Anti-Histone H3 (acetyl K36) antibody [EPR16992] (ab177179); Anti-KAT3B / p300 antibody [3G230 / NM-11] - ChIP Grade (ab14984)
Validation	c-Met Rabbit mAb #8198: Met (D1C2) XP® Rabbit mAb #8198. The exact sequence is proprietary,Entrez Gene: 4233 Human.<br>p-Met(Tyr1234/1235)Rabbit mAb #3077: Monoclonal antibody is produced by immunizing animals with a synthetic phosphopeptide corresponding to residues surrounding Tyr1234/1235 of human Met,Cooper, C.S. et al. Nature 311, 29-33.<br>AKT Rabbit mAb #4685: Monoclonal antibody is produced by immunizing animals with a synthetic peptide at the carboxyterminal sequence of mouse Akt, Franke, T.F. et al. (1997) Cell 88, 435-7.<br>Phospho-Akt (Ser473) Rabbit mAb #4060: Monoclonal antibody is produced by immunizing animals with a synthetic phosphopeptide corresponding to residues around Ser473 of human Akt, Franke, T.F. et al. (1997) Cell 88, 435-7.<br>p44/42 MAPK (Erk1/2) Rabbit mAb #4695: Monoclonal antibody is produced by immunizing animals with a synthetic peptide corresponding to residues near the C-terminus of rat p44 MAP kinase, Roux, P.P. and Blenis, J. (2004) Microbiol Mol Biol Rev 68, 320-44.<br>Stat3 (124H6) Mouse mAb #9139: Monoclonal antibody is produced by immunizing animals with a synthetic peptide corresponding to the sequence of human Stat3, Heim, M.H. (2001) J Recept Signal Transduct Res 19, 75-120.<br>Phospho-Stat3 (Tyr705) Rabbit mAb #9145: Monoclonal antibody is produced by immunizing animals with a synthetic phosphopeptide corresponding to residues surrounding Tyr705 of mouse Stat3, Heim, M.H. (2001) J Recept Signal Transduct Res 19, 75-120.<br>Anti-FOXO3A antibody - ChIP Grade (ab12162): Synthetic peptide: C-GLNVGNFTGAKQASSQ, with N-terminally added cysteine,conjugated to maleimide activated KLH, corresponding to amino acids 653-668 of Human FOXO3A, Entrez Gene: 2309 Human.<br>Anti-MGMT antibody [MT3.1] (ab39253): Recombinant full length protein corresponding to Human MGMT, Entrez Gene: 4255

Human.
Anti-Argonaute-2 antibody - ChIP Grade (ab32381): Synthetic peptide corresponding to Argonaute-2 aa 350-450 conjugated to keyhole limpet haemocyanin, Entrez Gene: 27161 Human.
Dicer (D38E7) Rabbit mAb #5362: Monoclonal antibody is produced by immunizing animals with a synthetic peptide corresponding to residues surrounding Ala1133 of human Dicer, Hutvágner, G. and Zamore, P.D. (2002) Science 297, 2056-60.
Anti-Histone H3 (acetyl K4) antibody [EPR16596] - ChIP Grade (ab176799): Synthetic peptide within Human Histone H3 aa 1-100 (acetyl K4).
Anti-Histone H3 (acetyl K9) antibody - ChIP Grade (ab4441): Synthetic peptide corresponding to Human Histone H3 aa 1-12 (acetyl K9) conjugated to Keyhole Limpet Haemocyanin (KLH), Ujfaludi Z et al. Coordinated activation of a cluster of MMP genes in response to UVB radiation. Sci Rep 8:2660 (2018).
Anti-Histone H3 (acetyl K27) antibody - ChIP Grade (ab4729): Synthetic peptide corresponding to Human Histone H3 aa 1-100 (acetyl K27) conjugated to Keyhole Limpet Haemocyanin (KLH), Festa BP et al. Impaired autophagy bridges lysosomal storage disease and epithelial dysfunction in the kidney. Nat Commun 9:161 (2018).
Anti-Histone H3 (acetyl K36) antibody [EPR16992] (ab177179): Synthetic peptide within Human Histone H3 aa 1-100 (acetyl K36).
Anti-KAT3B / p300 antibody [3G230 / NM-11] - ChIP Grade (ab14984): Recombinant full length protein (Human), Jian Y et al. Jade family PHD finger 3 (JADE3) increases cancer stem cell-like properties and tumorigenicity in colon cancer. Cancer Lett 428:1-11 (2018).

Eukaryotic cell lines

Policy information about cell lines

Cell line source(s)	Cell line sources are listed in "Cell lines and cell culture" section.
Authentication	The lines were authenticated by short tandem repeat (STR) profiling.
Mycoplasma contamination	The cell lines were tested negative for mycoplasma contamination.
Commonly misidentified lines (See ICLAC register)	N/A

Animals and other organisms

Policy information about studies involving animals; ARRIVE guidelines recommended for reporting animal research

Laboratory animals	Four-week-old female athymic BALB/c nude mice were used.
Wild animals	N/A
Field-collected samples	N/A
Ethics oversight	We have complied with all relevant ethical regulations for animal testing and research. All animal experiments were performed according to Health guidelines of Harbin Medical University Institutional Animal Use and Care Committee.

Note that full information on the approval of the study protocol must also be provided in the manuscript.

Flow Cytometry

Plots

Confirm that:

☒ The axis labels state the marker and fluorochrome used (e.g. CD4-FITC).

☒ The axis scales are clearly visible. Include numbers along axes only for bottom left plot of group (a 'group' is an analysis of identical markers).

☒ All plots are contour plots with outliers or pseudocolor plots.

☒ A numerical value for number of cells or percentage (with statistics) is provided.

Methodology

Sample preparation	The GBM cells were seeded into 6-well plates at a density of 1 ×105 cells per well in 1 mL fresh DMEM (supplemented with 5% FBS). After an incubation of 24 h, the culture medium of each well was replaced with 1 mL of fresh medium containing TMZ. After 3Days further incubation, the culture medium was removed and cells were washed two times with PBS buffer and detached by 0.02% (w/v) EDTA and 0.25% (w/v) trypsin solution, and then dispersed in 0.5 mL of PBS for flow cytometric measurement. Cells treated with DMSO were used as control.
Instrument	BD FACSCanto II.
Software	FlowJo software.
Cell population abundance	We didn't use the sorting function of flow cytomerter in the present study.

Gating strategy	The cell debris and dead cells were excluded by establishing the rational preliminary FSC/SSC gates.

☐ Tick this box to confirm that a figure exemplifying the gating strategy is provided in the Supplementary Information.
